# HIV-1 Integrates Widely throughout the Genome of the Human Blood Fluke *Schistosoma mansoni*

**DOI:** 10.1371/journal.ppat.1005931

**Published:** 2016-10-20

**Authors:** Sutas Suttiprapa, Gabriel Rinaldi, Isheng J. Tsai, Victoria H. Mann, Larisa Dubrovsky, Hong-bin Yan, Nancy Holroyd, Thomas Huckvale, Caroline Durrant, Anna V. Protasio, Tatiana Pushkarsky, Sergey Iordanskiy, Matthew Berriman, Michael I. Bukrinsky, Paul J. Brindley

**Affiliations:** 1 Department of Microbiology, Immunology & Tropical Medicine, and Research Center for Neglected Diseases of Poverty, School of Medicine and Health Sciences, The George Washington University, Washington, DC, United States of America; 2 Department of Microbiology, Faculty of Science, Mahidol University, Phyathai, Rachthewee, Bangkok; 3 Department of Pathology, Faculty of Medicine, Khon Kaen University, Muang Khon Kaen, Thailand; 4 Wellcome Trust Sanger Institute, Wellcome Genome Campus, Hinxton, Cambridge, United Kingdom; 5 Biodiversity Research Center, Academia Sinica, Taipei, Taiwan; 6 Veterinary Research Institute, Chinese Academy of Agricultural Sciences, Xujiaping 1, Lanzhou, Gansu, The People's Republic of China; 7 National Center for Biodefense and Infectious Diseases, School of Systems Biology, George Mason University, Manassas, Virginia, United States of America; University of Pennsylvania, UNITED STATES

## Abstract

Schistosomiasis is the most important helminthic disease of humanity in terms of morbidity and mortality. Facile manipulation of schistosomes using lentiviruses would enable advances in functional genomics in these and related neglected tropical diseases pathogens including tapeworms, and including their non-dividing cells. Such approaches have hitherto been unavailable. Blood stream forms of the human blood fluke, *Schistosoma mansoni*, the causative agent of the hepatointestinal schistosomiasis, were infected with the human HIV-1 isolate NL4-3 pseudotyped with vesicular stomatitis virus glycoprotein. The appearance of strong stop and positive strand cDNAs indicated that virions fused to schistosome cells, the nucleocapsid internalized and the RNA genome reverse transcribed. Anchored PCR analysis, sequencing HIV-1-specific anchored Illumina libraries and Whole Genome Sequencing (WGS) of schistosomes confirmed chromosomal integration; >8,000 integrations were mapped, distributed throughout the eight pairs of chromosomes including the sex chromosomes. The rate of integrations in the genome exceeded five per 1,000 kb and HIV-1 integrated into protein-encoding loci and elsewhere with integration bias dissimilar to that of human T cells. We estimated ~ 2,100 integrations per schistosomulum based on WGS, i.e. about two or three events per cell, comparable to integration rates in human cells. Accomplishment in schistosomes of post-entry processes essential for HIV-1replication, including integrase-catalyzed integration, was remarkable given the phylogenetic distance between schistosomes and primates, the natural hosts of the genus *Lentivirus*. These enigmatic findings revealed that HIV-1 was active within cells of *S*. *mansoni*, and provided the first demonstration that HIV-1 can integrate into the genome of an invertebrate.

## Author Summary

Schistosomiasis is a major neglected tropical disease (NTD), which afflicts > 200 million people in developing countries. The genome sequence of the schistosome parasite has been decoded; it includes > 10,000 genes. New approaches to control this NTD are sought and genomic information may provide targets for new treatments. Methods to determine the role and importance of specific genes would facilitate these tasks. The retrovirus HIV-1, the causative agent of HIV/AIDS, has been extensively studied and modified for use in biomedical research. Using a lab-modified form of HIV-1, we manipulated the genome of *Schistosoma mansoni*, one of the major species of schistosomes. Lab-modified HIV-1 infected schistosomes and inserted in the chromosomes of the parasite. These chromosomal insertions were mapped using next generation sequencing and were distributed throughout the chromosomes including the sex chromosomes. The findings were notable since they revealed that HIV-1 was active within cells of *S*. *mansoni*, and they provide the first demonstration that HIV-1 can integrate into the genome of an invertebrate. They pave a route forward for investigating new therapies for schistosomiasis.

## Introduction

Schistosomiasis is considered the most important helminthic disease of humanity in terms of morbidity and mortality, and is one of the major neglected tropical diseases (NTDs) [1–4]. Whereas > 90% of cases occur in Africa, where the major burden of disease lies, a recent outbreak in Corsica confirmed its re-emergence in Europe [5]. To accelerate discovery of intervention targets for schistosomiasis and to provide exploitable insights into the parasite biology and pathogenesis, concerted efforts are in train to produce reference genome sequences of the human schistosomes and related helminths [6–11]. To capitalize on the investment in flatworm poly-omics to identify novel control strategies, high-throughput systems for comprehensive studies of gene function have now become essential. However, because parasitic flatworms at large are difficult to maintain in the laboratory due to complex developmental cycles, they remain recalcitrant to genetic/cellular manipulations, presenting a significant bottleneck for adapting state-of-the-art approaches to elucidate gene function [12]. Current large-scale approaches, mainly involving medium-throughput RNAi screening [13, 14], currently provide a veneer only of information on gene function since the knowledge of characteristics and regulation of specific gene expression remains limited. To profoundly probe function at scale, protocols for routine manipulation of the genome need to be established and optimized; genes need to be disrupted, transgenes inserted, and expressed in a sustainable, and even tunable, fashion.

There has been progress in developing functional genomics for schistosomes and some other flatworms [12]. Vesicular stomatitis virus glycoprotein (VSV-G)-pseudotyped murine leukemia virus (MLV) was shown to transduce schistosomes, integrating the provirus into the chromosomes of *Schistosoma mansoni* [15, 16]. (Production of viruses with foreign viral envelope proteins is termed pseudotyping; pseudotyped is undertaken to increase host species and cell type tropism and/or enhance stability of the virions [17].) Germ-line transgenesis has been achieved by transducing *S*. *mansoni* eggs with VGV-G MLV, enabling the establishment of stable lines of transgenic parasites [16, 18]. In addition, eggs also might be transducible by pseudotyped human immunodeficiency virus-1 (HIV-1) [19]. Lentiviruses such as HIV-1 possess a desirable attribute for functional genomics, since these viruses can infect both dividing and terminally differentiated non-dividing cells; MLV can infect the former but not the latter [20, 21]. However, critical details are missing on the capacity of lentiviruses to integrate into chromosomes of flatworms and transcribe transgenes. In particular, given evolutionary divergence of flatworms and humans, the natural host HIV-1, it is necessary to characterize the preferred regions of integration before using lentiviral vectors for functional genomics. Here we report that infection of *S*. *mansoni* with pseudotyped HIV-1 lead to attachment of virions, reverse transcription of the RNA genome of HIV-1, and integrase-catalyzed insertion of the provirus into the genome of the blood fluke, and characterize the sites of integration. HIV-1-based manipulation of these parasites should enable advances in functional genomics for schistosomes and related platyhelminth pathogens.

## Results

### VSV-G-pseudotyped HIV-1 virions attach to surface of parasites

**Fig 1:**
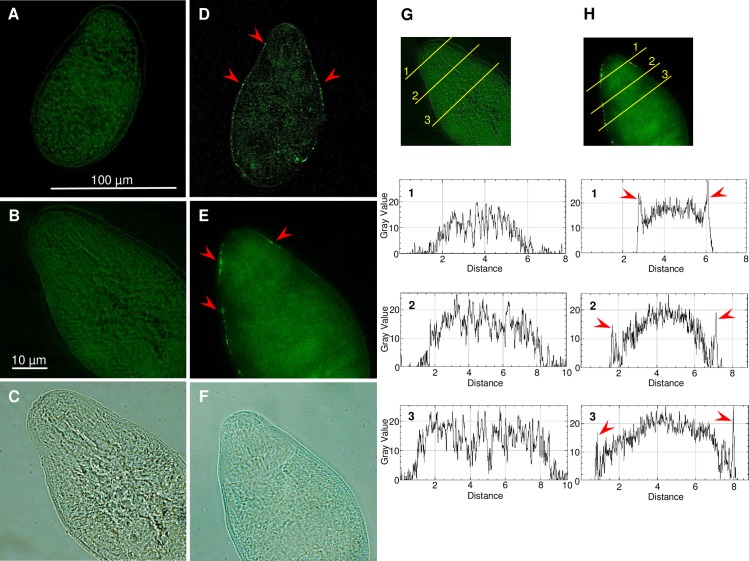
Localization of the vesicular stomatitis virus glycoprotein (VSV-G) pseudotyped HIV-1 virions on the surface of the schistosomulum of *Schistosoma mansoni*. **Panels A, B, C,** schistosomula exposed to culture medium containing 8 μg/ml polybrene for three hours, fixed and probed with anti-VSV-G rabbit polyclonal antibody and secondary Alexa Flour 488 chicken anti-rabbit IgG (control). **Panels D, E, F,** schistosomula exposed to VSV-G-pseudotyped HIV-1 in culture medium containing 8 μg/ml polybrene for three hours, fixed and probed as described in A-C. Micrographs captured with same exposure and magnification 40X and 63X; Red arrows indicate areas of high Alexa Flour 488 fluorescence within the schistosomulum tegument. **Panels G, H,** Gray value profiles of the cross-sections of images of the virus-exposed (H) and non-exposed (G) schistosomules. Red arrows indicate peaks of Alexa Flour 488 fluorescence on the tegument of the worm. Immunolocalization assays were undertaken at least twice for schistosomula and adult schistosomes.

The successful attachment of VSV-G pseudotyped HIV-1 to the tegument of schistosomes was demonstrated using an antibody specific for VSV-G. Specific binding was observed to the surface of both schistosomula (Fig 1A–1F) and adult worms (S1 and S2 Figs) following exposure to the virions. An evident fluorescence signal emitted by Alexa Fluor 488-labeled anti-VSV-G antibody was detected and measured using spinning disk confocal microscopy (Fig 1G and 1H). Moreover, the signal intensity observed mainly in the surface of the virion-transduced parasites significantly increased over three hours exposure (S2 Fig). These results demonstrated time-dependent attachment of the virions to the schistosome tegument. In addition, the binding pattern seen on the tegument of both the schistosomules and adult worms revealed a focal rather than general binding to the surface of this developmental stage (1D, 1E, S1E and S1F Figs). Schistosomes not exposed to virions and incubated with VSV-G primary antibody and schistosomes exposed to virions and incubated with the secondary antibody only did not exhibit fluorescence, thereby indicating specific binding by both the primary and secondary antibodies. Although, autofluorescence was evident in schistosomules and adult worms (Fig 1A and 1B and S1D Fig), that ‘fluorescence’ pattern was distinct and readily distinguished from the Alexa Fluor 488. signal (Fig 1H).

### HIV-1 RNA is reverse-transcribed in schistosomes and provirus integrated into the genome

**Fig 2:**
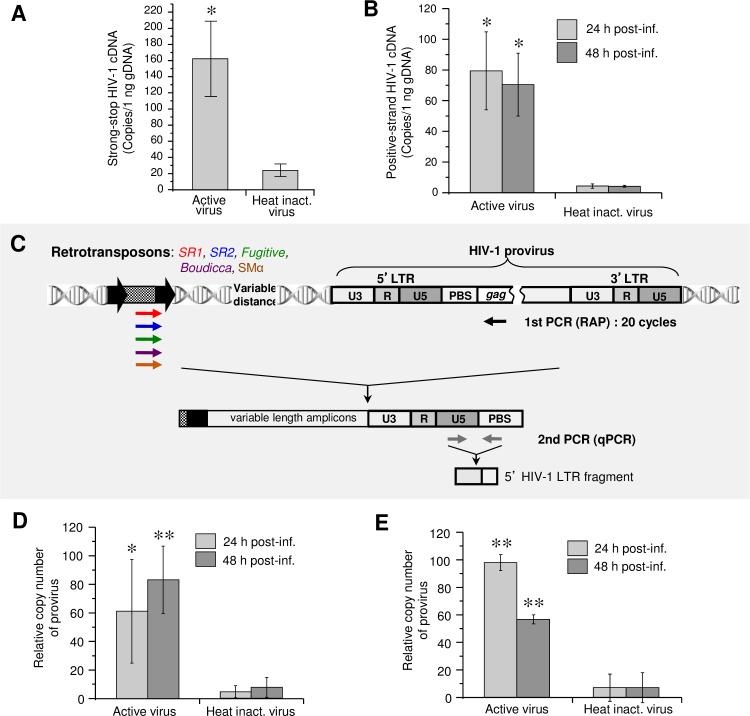
RNA genome of HIV-1 reverse transcribed in cells of *Schistosoma mansoni* and lentiviral cDNA integrated into the schistosome genome. **Panel A**. Quantitation of negative-strand, strong-stop HIV-1 cDNA in genomic DNA of schistosomula 24 hours after exposure to intact or heat-inactivated virions. **Panel B**. Quantitation of the late, positive-strand HIV-1 cDNA in schistosomula at 24 and 48 hours after incubation with virions. **Panel C.** Schematic representation of the nested two-step, quantitative retrotransposon-anchored PCR (qRAP) for relative quantitation of HIV-1 provirus integrated into the schistosome genome. Products of the first reaction using retrotransposon-specific primers were subjected to secondary PCR using provirus-specific primers. **Panel D**. Detection of HIV-1 provirus integrated into the schistosome genome using the primer set no. 1 specific for retrotransposons *SR1* and *SR2*. **Panel E**. Detection of HIV-1 provirus detected with primer set no. 2, specific for *fugitive*, *SMα*, and *Boudicca*. Statistical analysis: Student’s *t*-test; *, **—*P* ≤ 0.05, *P* ≤ 0.01 (active vs. heat-inactivated virions). The experiments were repeated ≥ three times.

Quantitative PCR (qPCR) of DNA extracted from schistosomula exposed to active or heat-inactivated virus was performed employing HIV-1 specific primers to estimate the copy number of HIV-1 cDNA molecules. Both early, strong-stop and late, positive-strand HIV- specific cDNAs were detected in parasites exposed to active HIV-1, whereas few copies were detected in parasites exposed to heat-inactivated virions (Fig 2A [early strong-stop; *P* ≤ 0.05, Student’s *t*-test] and 2B [late, positive-strand; *P* ≤ 0.01]). These findings established that reverse transcription of the RNA genome of HIV-1 had proceeded in the cells of virion-exposed parasites.

Thereafter, integration of HIV-1 cDNA into the schistosome genome was investigated. Integration of the provirus in human cells has been earlier assessed using a quantitative two-step *Alu*-based nested PCR [22]. We modified this approach to target multi-copy endogenous elements present in the schistosome genome; a method termed ‘quantitative Retrotransposon Anchored PCR’ (qRAP) [23]. Genomic DNA (gDNA) extracted from schistosomula was subjected to nested PCR employing a primer specific for the *gag* gene of HIV-1 in tandem with primers specific for several endogenous retrotransposons known from the genome of *S*. *mansoni* (Fig 2C). The relative copy number of integrated HIV-1 as estimated by qRAP was significantly higher in schistosomes transduced with active virions compared to the negligible signals from parasites exposed to heat-inactivated virions, at both 24 and 48 hours after transduction (Fig 2D and 2E). These findings indicated that HIV-1 cDNA reached the nuclei of schistosomes, and that the proviruses integrated into the genome of the parasite, at least in regions proximal to the endogenous retrotransposons employed as anchors for the qRAP [22]. Curiously, two inhibitors of reverse transcriptase, azidothymidine and nevirapine, each with a discrete mode of action, and an inhibitor of integrase, the diketo acid derivative 118-D-24, failed to block these events, as determined by qRAP targeting integration events (S3 Fig).

### HIV-1 integrates widely throughout the schistosome genome

In order to identify and map integrated HIV-1 proviruses within the reference genome of *S*. *mansoni* [6], an Illumina sequencing-based approach that utilized PCR to enrich for the integration events was adapted from a procedure named TraDIS (Transposon Directed Insertion-site Sequencing), which had been employed to characterize transposons in bacterial genomes [24]. The latter had been successfully adapted to localize integrations of VSVG-pseudotyped Murine Leukemia Retrovirus (MLV) in somatic and germ line-derived cells of schistosomes [16]. Illumina libraries were prepared from genomic DNA and then amplified to enrich for integration sites (S1 and S2 Tables). High throughput sequencing of the TraDIS libraries constructed from both the 5- and 3’long terminal repeats (LTRs) of HIV-1 yielded >25,000 paired sequence reads with HIV-1 start sites from libraries constructed from both the 5’- and 3’-long terminal repeats (LTRs) of the lentivirus. About ~8,000 integrations were identified (Table 1), comprising 1,827 and 6,258 non-redundant events from the 5’- and 3’-LTR libraries, respectively. Of these sites, most were unique clusters where neighboring integrations were separated by > 250 bp.

**Table 1:** Numbers of integrations catalyzed by integrase or other molecular process, identified in the genome of *Schistosoma mansoni* exposed to pseudotyped HIV-1 virions. TraDIS identified the integrations, which were mapped to the draft genome of *S*. *mansoni*.

	HIV-1 integrations^a^		
	Integrase-catalyzed (%)	Process unresolved (%)	Total
*HIV 5’-end library*			
Merged reads^b^	134 (15)	762 (85)	896
Read pairs^c^	119 (12)	851 (88)	970
Unique positions^d^			1,827
Unique clusters^e^			1,753
** ** *HIV 3’-end library*			
Merged reads	357 (10)	3,184 (90)	3,541
Read pairs	84 (3)	2,736 (97)	2820
Unique positions			6,258
Unique clusters			6,065

^a^ Integrase-catalyzed: events where the terminal CA residues of the HIV-1 LTR located immediately adjacent to mapped region of the schistosome genome. Integrase catalyzes both double stand cleavage and strand transfer steps that integrate the provirus into the genome [43]. Process unresolved: whereas some of these events may be integrase-catalyzed, others may have resulted from homologous recombination or from non-homologous end joining [47, 48]. Sequence complexity at the extremity of the aligned region frequently thwarted precise definition of integration boundary.

^b^ Merged reads: two reads sequenced that together were longer than the DNA fragment; hence overlapping portions could be aligned.

^c^ Read pairs: reads that cannot be overlapped because the DNA fragment presumably was longer than 200 bp.

^d^ Unique positions: Discrete reads with exact integration positions were counted only once.

^e^ Unique clusters: Neighboring unique integration positions within 250 bp were considered as one cluster.

**Fig 3:**
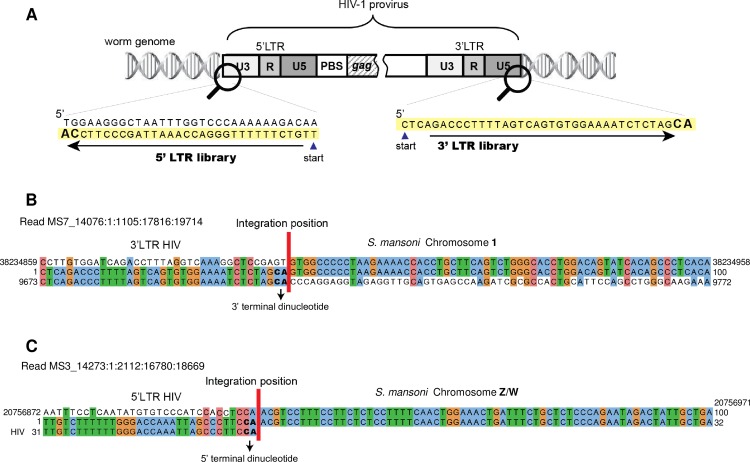
Modified Transposon Directed Insertion-site Sequencing (TraDIS) recovered integrated HIV-provirus. **Panel A**. Schematic of HIV provirus within the schistosome genome highlighting the termini of the 5’- and 3’-LTRs. **Panels B, C**. Representative integration boundaries (vertical red line) between the 3’-LTR- or 5’-LTR-ends of the provirus and schistosome genome within chromosome 1 and Z/W, respectively. The provirus dinucleotides CA at the 3’-LTR and TG at the 5’-LTR (CA in the reverse complementary to the 5’-LTR as indicated in Panel A) are highlighted. In the multiple sequence alignment, the top line is the sequence of the schistosome genome, the middle line is the 100 bp-sequence of the indicated TraDIS reads, and the bottom line shows the sequences at the termini of the 3’-LTR (B) or 5’-LTR (C) of HIV-1 clone pNL4-3 [69] employed to infect the schistosomes. Coordinates are indicated at the ends of the alignment.

Mapping the integrated proviruses to the schistosome reference genome revealed a broad distribution of integrations throughout all eight chromosomes of the parasite, comprising chromosomes 1 to 7 and Z/W, the sex chromosomes. Notably, integrations into the mitochondrial genome were also mapped (Table 2). Similar findings were apparent from analysis of sequences mapped from either the 5’-LTR- or 3’-LTR-end libraries. It was evident that numerous integrations of HIV-1 provirus into the schistosome genome had been catalyzed by integrase, given the presence of the diagnostic dinucleotides CA at the 3’-LTR and TG at the 5’-LTR termini of HIV-1 at the integration junctions immediately flanking the schistosome genome (Fig 3A and S1 Table) (S4 Fig). Fig 3B and 3C present representative integration boundaries (vertical red bar) between the 3’-LTR termini of the provirus and the schistosome genome within the chromosome 1, or 5’-LTR-ends and chromosome Z/W, respectively. Sequences of a series of junctions of additional, representative integrations recovered using TraDIS from the 3’-LTR-end library are shown in S4 Fig.

**Table 2:** Numbers of HIV-1 proviruses integrated across the nuclear, the autosomes and the Z/W sex chromosomes, and mitochondrial genomes of *Schistosoma mansoni*

Chromosome	Assembly length	5’-LTR integrations	3’-LTR integrations
**1**	79,986,414	405 (5.1) ^a^	1,363 (17.0)
**2**	38,216,055	186 (4.9)	710 (18.6)
**3**	38,028,725	189 (5.0)	676 (17.8)
**4**	34,586,772	176 (5.1)	594 (17.2)
**5**	10,324,086	51 (4.9)	181 (17.5)
**6**	20,039,393	107 (5.3)	332 (16.6)
**7**	11,043,263	61 (5.5)	180 (16.3)
**Z/W**	61,504,481	279 (4.5)	930 (15.1)
**Mitochondrion**	19,871	8 ^b^	17 ^b^
**Unplaced**	70,778,700	291 (4.1)	1,082 (15.3)

^a^ Parenthesis: number of integrations per Mb

**Fig 4:**
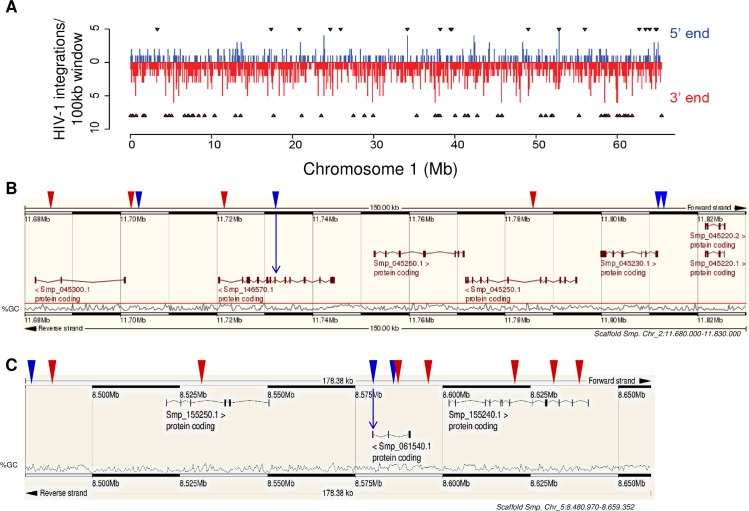
Integrations within the genome of schistosomula infected with HIV-1. **Panel A.** Schematic region within chromosome 1 (Chr 1) of *S*. *mansoni* indicating the number of integration events in contiguous 100 kb sections along the chromosome (~65 MB). Integrations recovered from the 5’- and 3’-LTRs-end libraries are shown in blue and red, respectively. Arrowheads indicate HIV-1 integrase-catalyzed events (see Table 1). **Panels B, C.** Representative schistosome loci targeted by integrase-driven HIV-1 in chromosomes 2 and 5, respectively. Integrations from the 5’- and 3’ LTR-end are shown with blue or red triangles, respectively. Arrows indicate integrations into exons of protein-coding genes, i.e. Smp_146570.1 in chromosome 2 (Panel B) and Smp_061540.1 in chromosome 5 (Panel C). Genome coordinates of integration events indicated here were: chromosome 2 (11680000 to 11830000) 5’-LTR library: 11703778, 11734288 (exon 10 of Smp_146570), 11812331, 11812694, 3’-LTR library: 11682679, 11701658, 11721703, 11786909, chromosome 5 (8480974 to 8659352) 5’-LTR library: 8480974 (exon 6 of Sm_061540), 8580018, 8586055, 3’-LTR library: 8484589, 8529907, 8580599, 8594743, 8621686, 8632719, 8638819, 8659352. Schematics provided in panels B and C were obtained and modified from WormBase ParaSite, http://parasite.wormbase.org/ [83].

Proviruses of HIV-1 distributed throughout the eight pairs of chromosomes of *S*. *mansoni*. A frequency distribution of integration events along the entire ~65 Mb length of chromosome 1 (Chr 1) illustrated the rate of integrations throughout the nuclear genome as recovered using TraDIS. Some regions represented integration hotspots with a rate exceeding five integrations of HIV-1 provirus per 100 kb of chromosome. HIV-1 integrase-mediated events are indicated with arrowheads above and below the windows; here the dinucleotides CA and TG were characterized in the sequenced analysis at the termini of the integrated 3’-LTR and 5’-LTR of the provirus, respectively (Fig 4A), evidence of catalysis by HIV-1 integrase [25]. Examples of integration events within chromosomes 2 and 5, as detected in the 5’-end LTR and 3’-end LTR libraries, are shown in Fig 4B and 4C. The event characterized in chromosome 2 lies within exon 10 of Smp_146570, a histidyl-tRNA synthetase-related protein (Fig 4B), and that in chromosome 5 lies in exon 6 of Smp_061540, an amino acid transporter homologue (Fig 4C). The genes were inferred by protein orthology and coordinates of the integration events are provided (Fig 4 legend). Note also numerous other integration sites, the positions of which are indicated with the arrowheads; blue colored arrowheads indicate events detected in the 5’-LTR-end library and red-colored in the 3’-LTR-end library (Fig 4B and 4C).

### Integration bias for non-coding regions

**Fig 5:**
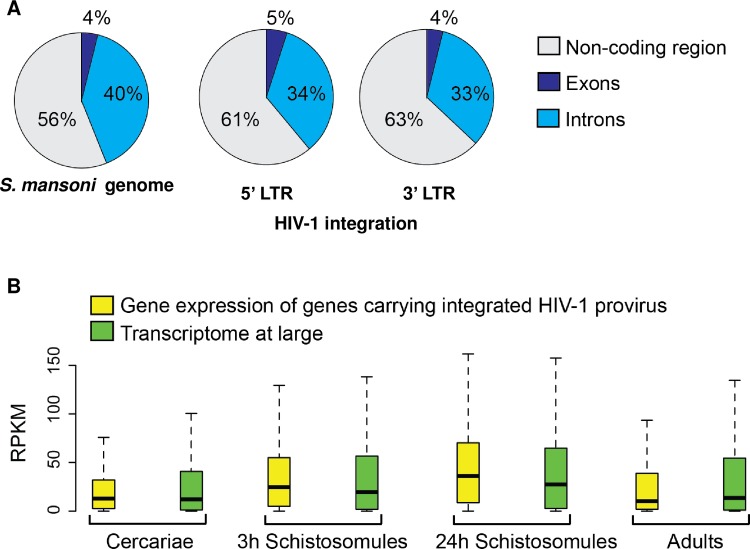
Genome wide distribution of HIV-1 integrations. **Panel A**. Left, pie chart showing the percentages of the indicated regions in the genome of *S*. *mansoni*. Center, pie chart showing the percentages of HIV-1 integrations recovered from the 5’-LTR-end library classified according to the function of the mapping site. Right, pie chart showing the percentages of HIV-1 integrations from the 3’-LTR-end library classified according to the function of the mapping site. **Panel B**. Box plots showing expression of genes carrying integrated HIV-1 provirus (yellow) and transcriptomes at large (green) of several developmental stages. The lines extending parallel from the boxes (commonly referred as “whiskers”) indicate variability outside the upper and lower quartiles of the set of values. The upper and the lower limits of the whisker indicate the upper and lower extreme values, respectively. RPKM, reads per kilobase per million mapped reads.

Further analysis of the integration events revealed that exons contained 4% of the integrations, introns contained ~34.5%, whereas ~62% were detected within non-coding regions. By comparison, 4, 39 and 57% of the *S*. *mansoni* genome is composed of exons, introns, and non-coding regions, respectively (Fig 5A). Despite the apparent concordance of integration frequencies with genome composition of exons, introns and non-coding regions, statistical analysis revealed a significant tendency of proviral HIV-1 to integrate into non-coding regions (binomial proportion one-tailed test, *P* ≤ 0.01); this was dissimilar to MLV, which did not show bias for any particular region [16].

Given the availability of a curated RNA-seq database from discrete developmental stages [6], we compared expression levels of genes where HIV-1 integrations were identified to the transcriptomes at large of cercariae, schistosomula at three and 24 hours following mechanical transformation from cercariae, and adults. Bias was not evident for integration of HIV-1 into genes actively transcribed at these developmental stages (Fig 5B).

### Whole genome sequencing reveals several integrations per schistosome nucleus

Given that both the qRAP and TraDIS approaches revealed that HIV-1 provirus had integrated widely into the schistosome genome, we undertook Whole Genome Sequencing (WGS) to precisely quantify integrated HIV-1 proviruses. Following WGS of genomic DNA from virion-exposed schistosomules to a depth of 48X coverage, Illumina reads were aligned to the genomes of both *S*. *mansoni* and HIV-1. Alignments were curated to remove false positive integrations and reads entirely of schistosome origin. Reads were assigned to mapping categories according to their position in either genome: 1) sequence reads containing HIV-1 adjacent to schistosome sequence, i.e. integration site within the read; 2) independent, i.e. pairs of sequence reads with one read aligned to the schistosome genome and the other to HIV-1; and 3) read pairs that matched only HIV-1, i.e. reads solely of lentivirus origin (S5A Fig and S4 Table). The WGS data revealed 82 reads among the three categories, and 60 integrations, 35 and 25 events within categories 1 and 2, respectively (S5A Fig). S5B Fig, presents a representative alignment of reads mapped to the genomes of *S*. *mansoni* and HIV-1, i.e. sequence reads containing partial HIV-1 sequence and partial schistosome sequence. This particular HIV-1 integration event, within the ZW chromosome, was one of 35 events in category 1 (above) (S5 Fig).

Subsequently, numbers of integrations per worm were estimated. The WGS library was constructed using 1,700 ng of genomic DNA from ~5,000 schistosomula exposed to virions. Given the diploid genome of *S*. *mansoni* has an estimated mass of 0.79 picogram [6], the total number of nuclei in the WGS was ~2.2 x 10^6^. WGS was performed to a depth of sequence coverage of ~48 haploid genome-equivalents, resulting in 207 million paired (and properly mapped) reads among which 60 integrations were detected. Given that a diploid genome is equivalent to 7.29 million reads, the number of expected integrations per nucleus is 60 x 7.29/207 = 2.1 integrations (95% confidence intervals 1.6, 2.7), representing ~2,100 integrations per schistosomulum (it has been estimated that the 48 hour-old schistosomulum comprises ~1,000 nuclei [26]).

## Discussion

This report characterizes the integration of HIV-1 provirus within the chromosomes of *Schistosoma mansoni*. It also provides, to our knowledge, the first demonstration of integration of HIV-1 into the genome of an invertebrate. Inoculation of cultures of schistosomes with HIV-1 led to attachment of virions to the surface of the blood fluke, reverse transcription of the RNA genome of HIV-1, and integration of the provirus into the genome. Notwithstanding that transduction of these parasites was facilitated by pseudotyping virions with VSV-G, and that transduction proceeded in the absence of CD4 and other cellular receptors expressed on activated T and other receptive cells, biochemical processes that evolved for parasitism of primates [27] by lentiviruses proceeded within the cytoplasm and nuclei of schistosome cells, including reverse transcription of the RNA genome, assembly of a pre-integration complex, transit to the chromatin and integrase-catalyzed integration of the provirus into the chromosomes.

Lentiviruses traverse the nuclear envelope, and consequently HIV-1 can productively transduce non-dividing target cells, such as macrophages. By contrast, gammaretroviruses such as MLV cannot transverse the nuclear envelope; rather, MLV accesses the chromosomes at mitosis following dissolution of the nuclear membrane [28, 29]. HIV-1 possesses a conclusive advantage over MLV for functional genomics since it can infect both dividing (as can MLV) and terminally differentiated non-dividing cells (which MLV cannot) [20, 21]. For example, HIV can transduce non-dividing human cells, including stem cells, prior to differentiation, and terminally differentiated cells, such as monocyte-derived macrophages, astrocytes and microglia [30, 31]. The pre-integration events in the developmental cycle of HIV are essentially discrete from the events of the MLV life cycle: the pre-integration complex (PIC) of HIV mixes with chromatin after transit through the intact nuclear envelope whereas the MLV PIC mixes with chromatin during mitosis [32]. Whether the PIC of HIV can integrate in the chromosomes without passage through the nuclear envelope remains to be determined but this seems unlikely given the dissimilarity in structure of the PICs of HIV and MLV. It is feasible that HIV-1 entered the nuclei of *S*. *mansoni* cells during interphase, and integrated into non-dividing cells [33]. HIV-1 also may access the chromosomes at mitosis although evidence that this mode of replication leads to productive infection is not yet available. Nonetheless, one or both of these routes may have led to the widespread integration of HIV-1 in the schistosome genome.

In view of earlier demonstrations of transduction of schistosomes by MLV [15, 16] and the present findings with HIV-1, it is clear now that pseudotyped retroviruses can accomplish chromosomal integration and vertical transmission in schistosomes. Moreover, infection by HIV-1 appears to be efficient: based on titers of virions to which these parasites were exposed and, assuming the presence of ~10^4^ virion particles in one pg/ml of p24CA (the major structural protein of the HIV-1 virion capsid; there are ~1,000 p24CA proteins in the mature virion) [34], each schistosomulum was exposed to ~0.5 x 10^6^–1x10^6^ virions. Since the WGS analysis detected ~2,100 integrations per schistosomulum, we estimated an integration efficiency of ~0.25–0.5%. Spinoculation, centrifugation of the worms in the presence of virions [developed for human T cells [22, 35], delivered >5-fold increase of viral entry and increased numbers of integrations (S6 Fig). It should be noted that natural infection of schistosomes by HIV-1 in people co-infected with both pathogens is highly unlikely to occur given that schistosomes do not express HIV-1 receptors. In this study, the HIV-1 virions were pseudotyped with G protein of vesicular stomatitis virus (VSV-G), which binds with a highly ubiquitous LDL family receptors (LDLR) and endows the pseudotyped virus with pantropism [36]. This process does not happen in natural conditions. This is supported by the lack of HIV-1 sequences in the curated genome of *S*. *mansoni* [6, 8], which represents an established laboratory strain of *S*. *mansoni* that is maintained in rodents [37].

Given the mechanism of productive infection by HIV-1 of CD4^+^ T lymphocytes, events in schistosomes would have taken place in concert with cellular factors. Some processes, including endogenous reverse transcription, can proceed *in vitro* outside host cells [38–40], and perhaps proceeded here in the presence of a minimal number of human cellular factors incorporated into the virion during virion production. However, chromosomal integration and other processes require specific factors in human cells, suggesting that orthologues or even less conserved surrogates from schistosome were adequate. Homologues of factors that contribute to establishment of infection appear to be present in *S*. *mansoni* (S5 Table), including a capsid-binding protein (Smp_094810), reverse transcription complexes-interacting protein (Smp_100090), importins involved in the nuclear translocation of the viral pre-integration complex e.g. Smp_051210, Smp_142770, and integrase-interacting protein (Smp_125050). However, HIV-1 exhibited divergent integration preference in schistosomes compared to T cells, a site preference influenced by lens-epithelium-derived growth factor, LEDGF/p75. LEDGF/p75 stabilizes and tethers the intasome, a tetramer of integrase proteins bridging the termini of the provirus, to the nucleosome [41, 42]. Absence of an orthologue of LEDGF/p75 may account for discrete integrations profiles between schistosome and human cells.

Integrase-dependent integration of full-length proviruses took place; the intact termini of the LTRs provided cogent evidence of catalysis by integrase [43]. Recovery of twice as many events from the 3’-LTR library suggested that mutations of the provirus also occurred such that rearrangements, truncations or deletions of the HIV-1 proviral genome influenced the efficiency of the TraDIS targeting the 5’-LTR. These phenomena occur during HIV-1 infection [44–46], but may be less surprising in the exotic setting of tissues of a schistosome. Truncated versions of MLV occur in schistosome chromosomes [15, 16]. Variants of truncated HIV-1 genomes and integration junctions that lacked CA residues at the termini of the HIV-1 provirus suggested integrase-independent recombination of incomplete reverse transcripts. These integrations may have resulted from homologous recombination or from non-homologous end joining [47, 48]. The entire lentiviral cDNA containing the central flap, a plus strand overlap in the 3’-terminus of *pol*, has an advantage over truncated cDNAs for importion into the nucleus [49, 50]. Yet completion of reverse transcription did not appear to be essential for insertion of viral cDNA into the schistosome genome. Perhaps HIV-1 exploited an alternative nuclear import mechanism in schistosomes. Numerous integrations of 3’-end HIV-1 cDNAs, which may represent intermediate products of reverse transcription, supported this possibility. In like fashion, truncated forms of integrated HIV-1 provirus that appear to represent products of incomplete reverse transcription frequently occur during HIV-1 infection in humans [51]. Since spinoculation enhanced contact of virions with the surface of the schistosome, the virions may have transduced the tegument rather than deeper tissues. The tegument of blood stream forms of the schistosome is syncytial, with multiple nuclei that are not in cell cycle synchrony [52, 53]. This arrangement may have facilitated contact of provirus with chromosomes, and recombination of incomplete reverse transcripts of HIV-1 into the genome.

Integrated proviruses preferred less gene rich regions but nonetheless proviruses were distributed in both coding and non-coding regions in the genome of *S*. *mansoni*. Within the Retroviridae, species-specific preferences for sites of integration have evolved so that, for example, HIV-1 prefers actively transcribed genes in euchromatin whereas MLV exhibits bias for promoters of actively transcribed genes, enhancers and CpG islands [54]. Indeed, half the MLV integrations occur within < 2% of the genome of some human cell lines [55]. By contrast, the distribution of HIV-1 integrations in schistosomes was reminiscent of site selection in human chromosomes by prototype foamy virus, a spumavirus, and the *Alphavirus* avian sarcoma leukosis virus (ASLV) [56]. ASLV prefers open euchromatin but does not show bias for transcriptional elements [57]. Other differences were seen including the inactivity of inhibitors of reverse transcriptase and integrase in schistosomes and integration into the mitochondrial genome. The inactivity of integrase inhibitor 118-D-24 on HIV-1 integration seemed consistent with the finding that numerous integrations may have occurred independent of insertion by integrase. However, the same or higher efficiency of reverse transcription in the worms treated with azidothymidine and nevirapine, suggested distinctive mechanisms of elimination of all these compounds, the inhibitors of reverse transcription and integrase, from schistosome cells. This might have been accomplished by aquaporins and other transporters active in schistosomes [58, 59]. Moreover, the inhibitors may not have entered schistosome cells that had been infected with HIV-1. Integration into the mitochondrial genome was notable given that this phenomenon may not have been reported in HIV-1 infected human tissues, as indicated by absence of reports of this type of event within the Retrovirus Integration Database, a public database for retroviral insertion sites [60].

A compelling attribute of HIV-1 versus gammaretroviruses such as MLV is the ability of the former to efficiently infect both dividing and terminally differentiated cells. Here we tested the HIV-1 transduction in the schistosomula and adult worms where many cells are differentiated and do not proliferate. Since we sought to investigate reverse transcription and chromosomal integration, access to substantial quantities of RNA and DNA from the parasites facilitated these analyses. Therefore, it was less challenging to investigate these biochemical processes of HIV-1 in blood stage schistosomes, schistosomula and adults (rather than eggs). In future studies we plan to investigate the feasibility of deriving transgenic lines of schistosomes by transducing eggs with VSVG-HIV-1, given that eggs of schistosomes have been successfully transduced with pseudotyped MLV [61]. Eggs of *S*. *mansoni* have been exposed also to pseudotyped HIV-1 virions carrying transgenes encoding microRNA-adapted short hairpin RNAs targeting genes expressed in schistosome eggs. The HIV-exposed eggs were inoculated into the pulmonary circulation of mice, which led to phenotypic changes in the inflammatory response, presumably following gene knockdown [19].

Establishment of transgenic lines of schistosomes, derived from retrovirus- and lentivirus-transduced eggs, expressing transgenes including Cas9 nuclease, should now be achievable. This would enable generation of specific knock-out lines using CRISPR-Cas9 gene editing as recently demonstrated for a parasitic nematode [62] and induction of stable gene knock-down from expression of shRNAs from integrated transgenes delivered using lentiviral vectors [14, 19, 63]. The performance of *cis*-regulatory elements to drive the expression of transgenes, insulator elements to prevent chromatin silencing position effects, and the use of selection markers need to be further investigated. How the widespread integration of HIV-1 in the schistosome chromosomes influences gene expression awaits investigation, as does the impact of the integration bias. The high-throughput approaches employed here to estimate the number of integration events investigated genomic DNAs pooled from large numbers of schistosomules, and hence the relatively high number of integrations represents what took place within the population of pooled HIV-transduced parasites. Transducing schistosomes with high titers of HIV-1 may also facilitate insertional mutagenesis-based forward genetics. Manipulation by transgenesis, knockout and/or gene editing by CRISPR/Cas 9-related approaches [64, 65] can be predicted to enhance understanding of these pathogens, their somatic stem cells [66], reproduction, longevity in infected hosts [10, 67], and intervention targets. These approaches may also facilitate establishment of sex-biasing gene drives to block the spread of schistosomiasis [68].

To summarize and conclude, this report presents enigmatic findings that reveal that certain steps of HIV-1 replication are active within cells of the human blood fluke, *Schistosoma mansoni*, a parasitic flatworm responsible for the major neglected tropical disease (NTD) schistosomiasis. Facile manipulation of schistosomes using lentiviruses should enable advances in functional genomics in these and related NTD pathogens including tapeworms, in particular concerning their non-dividing cells. Such approaches have hitherto been unavailable, and the lack of these kinds of tools underpins why the NTDs are neglected: the helminth NTD pathogens have not been readily tractable to laboratory investigation. Unlike the retrovirus MLV, which we investigated previously [15, 63], HIV-1 integrates into non-dividing cells. This represents a distinct advantage in applications such as transient transformation of adult and schistosomula stages, and thus is a substantial advancement in the functional genomics of these important parasites. A corollary of the findings is that lentiviral pre-integration complexes exploit either evolutionary conserved mechanisms or that HIV-1 can employ diverse strategies of nuclear import and integration. Although HIV-1 has been considered as a specialist virus because it uses species-specific receptors for host cell entry, the new findings suggest, rather, that it is generalist in use of intracellular pathways at post-entry steps of infection.

## Methods

### Ethics statement

Mice experimentally infected with *S*. *mansoni*, obtained from the Biomedical Research Institute, Rockville, MD were housed at the Animal Research Facility of the George Washington University Medical School, which is accredited by the American Association for Accreditation of Laboratory Animal Care (AAALAC no. 000347) and has an Animal Welfare Assurance on file with the National Institutes of Health, Office of Laboratory Animal Welfare, OLAW assurance number A3205-01. All procedures employed were consistent with the Guide for the Care and Use of Laboratory Animals. Maintenance of the mice and recovery of schistosomes were approved by the Institutional Animal Care and Use Committee of the George Washington University.

### Production of VSV-G pseudotyped HIV-1 virions

To produce vesicular stomatitis virus glycoprotein–pseudotyped HIV-1 (VSV-G-HIV-1) virions for transduction of schistosomes and analysis of reverse transcription, nuclear import and integration, HEK293T (ATCC, Manassas, VA) cells were co-transfected with HIV-1 proviral clone pNL4-3 (T cell-tropic HIV-1 subtype B isolate) [69] and the pcDNA-VSV-G plasmid at a 4:1 ratio using Metafectene as described [70]. The resulting viruses were harvested 48 h later, passed through a 0.45 μM diameter pore size membrane and then incubated in 10 mM MgCl_2_ and 50 U/ml of RNase-free DNase I (Roche, Indianapolis, IN) at 37°C for two hours. Thereafter, virus particles were concentrated by centrifugation (Beckman SW-28 rotor, 100,000x*g*, 24,000 rpm) for two hours at 4°C through a sucrose (30% in PBS) cushion. Pellets of virions were re-suspended in DMEM after which the activity of reverse transcriptase (RT) [71] and concentration of HIV-1 p24 antigen (as measured using the Alliance HIV-1 p24 Antigen ELISA kit, Perkin Elmer, Waltham, MA) were determined in order to estimate the virion titer. Schistosomes were transduced with virions at titers ranging from 0.5 to 1 μg capsid p24CA per ml. Although the NL4-3 virus cannot replicate in schistosome cells because of its tropism for human T cells, nonetheless the studies undertaken here were performed under BSL2 containment.

### Schistosomes

Schistosome-infected mice and infected *Biomphalaria glabrata* snails were provided by the NIAID Schistosomiasis Resource Center at the Biomedical Research Institute (Rockville, MD) through NIH-NIAID Contract HHSN272201000005I for distribution through BEI Resources. Schistosomula were obtained by mechanically transformation of cercariae released from infected *B*. *glabrata* snails as described [72]. Briefly, cercariae were concentrated by centrifugation (2,000 rpm/10 min) and washed 3 times in schistosomula wash medium (DMEM supplemented with 2% penicillin, streptomycin, fungizone, and 10 mM HEPES) [72]. Cercarial tails sheared off by repeated passes through a 22G emulsifying needle were removed by Percoll gradient centrifugation, schistosomula were washed three times in schistosomula wash medium and cultured at 37°C under 5% CO_2_ in air in modified Basch’s medium [72]. Adult schistosomes were recovered from mice by portal perfusion, washed in 1x PBS supplemented with penicillin, streptomycin and fungizone, and cultured as described [72].

### Transduction of schistosomes with pseudotyped HIV-1

Schistosomula (~10^3^–10^4^) were cultured in 24-well tissue culture plates in one ml of modified Basch’s medium [72] for one day after transformation. Thereafter, the culture medium was replaced with 500 μl of intact or heat-inactivated (2 hours, 65°C) VSV-G pseudotyped HIV-1 virions, 500 μl of schistosomula medium and 8 μg/ml of the cationic polymer polybrene [16] to a final volume of one ml. The plate was subjected to centrifugation with the virus (1,000 x *g*, 60 min, 23°C), i.e. spinoculation [22], and 24 hours later the culture medium was replaced with fresh medium. Schistosomula were harvested at 24 and 48 hours after incubation with the VSV-G pseudotyped HIV-1 virions, washed 3 times with 1x PBS, snap froze in dry ice, and stored at -80°C. In some experiments the schistosomula were cultured in the presence of the virions without spinoculation. Spinoculation resulted on 5- to 6-fold increase on reverse-transcription and genome integration efficiency measured by qPCR and qRAP, respectively (S6 Fig).

### Immunolocalization of VSV-G on surface of schistosomes

Schistosomula and adult worms were exposed to VSV-G pseudotyped virions in the presence of polybrene. At intervals from 0 to three hours, the presence of VSV-G pseudotyped virions attached to parasites was investigated. In brief, at indicated time points the culture medium was removed, schistosomes were washed 3X with Tween-20 in PBS (PBS-T) to remove unattached virions, after which formaldehyde fixation was undertaken for one hour to cross-link virions bound to the parasite surface. Fixed schistosomes were permeabilized in 0.2% Triton X-100 in PBS for 15 min and washed with PBS-T for 5 min, 3 times. Non-specific epitopes were blocked by incubating schistosomes overnight in 5% normal horse serum in PBS and thereafter probed with primary antibody, rabbit anti-VSV-G antibody (Sigma-Aldrich, St. Louis, MO) diluted 1:500 in PBS for two hours at room temperature. The parasites were washed for 5 minutes 3 times with PBS-T, probed with secondary antibody, Alexa Fluor 488 chicken anti-rabbit antibody (Life Technologies, Frederick, MD) diluted 1:500 in PBS, washed with PBS-T and mounted on slides with Fluoromount-G (Southern Biotech, AL). The schistosomes were examined using a Zeiss Axio Observer A.1 inverted microscope fitted with an AxioCam ICc3 camera (Zeiss) and/or Zeiss 710 Cell Observer spinning disk confocal laser scanning microscope. Micrographs were captured with a 10X objective for adult schistosomes, 40X and 63X objectives for schistosomula. Manipulation of digital images was undertaken with the assistance of AxioVision 4.6.3 software (Zeiss), with manipulations were limited to insertion of scale bars, adjustments of brightness and contrast, cropping and the like. Image enhancement algorithms were applied in linear fashion across the entire image and not to selected aspects. The intensity of the fluorescence from schistosomes exposed to HIV-1 virions was quantified using ImageJ, https://imagej.nih.gov/ij/. In brief, 20 sections of the same area were selected at random for each micrograph, 10 sections of the background and 10 sections within the parasite, and the signal intensity for each obtained. The mean and standard deviation of signal intensities were determined for each panel, and the ratio of parasite signal intensity to the background signal calculated.

### Activity of HIV-1 reverse transcriptase in schistosomes

Total genomic DNA was isolated from active- or heat-inactivated lentivirus-transduced schistosomula harvested 24 or 48 hours after transduction, using the AquaPure system (Bio-Rad, Hercules, CA), and employed as template for qPCR targeting the negative-strand strong-stop and the positive-strand HIV-1 cDNA [73, 74]. Briefly, sequences of the primers amplifying the strong-stop DNA were: forward primer M667 (5’-GGCTAACTAGGGAACCCACTG-3’), reverse primer AA55 (5’-CTGCTAGAGATTTTCCACACTGAC-3’), and Taqman probe Er-LTR (5’-FAM-GTCACACAACAGACGGGCACACACTA-TAMRA-3’) specific for the R-U5 region of the LTR of HIV-1. The second set recognizes the positive-strand DNA (late reverse transcription product) and consisted of primers: FOR-LATE (5’-TGTGTGCCCGTCTGTTGTGT-3’), REV-LATE (5’-GAGTCCTGCGTCGAGAGATC-3’), and Taqman probe Lt-LTR-Prb (5’-FAM-CAGTGGCGCCCGAACAGGGA-TAMRA-3’) specific for the U5-Ψ LTR region. Quantitative PCRs were performed in triplicate, using 96-well plates (Bio-Rad), with a denaturation step at 95°C, 3 min followed by 40 cycles of 30 sec at 95°C and 30 sec at 55°C, using a real-time thermal cycler (iCycler, Bio-Rad) fitted with the Bio-Rad iQ5 detector. Reactions were carried out in volumes of 20 μl with 0.3 μM primer-probe sets, Perfecta qPCR FastMix, UNG (Quanta Bioscience, Gaithersburg, MD) and 100 ng of total DNA isolated from active- or heat-inactivated HIV-1-transduced schistosomula as template. Ten-fold serial dilutions of DNA from 8E5 (derivative of CEM) cells, a human T lymphoblastoid line that contains a single copy of HIV-1 LAV provirus per cell, was used as the quantitative standard [75]. Findings are presented as copy numbers of negative-strand (strong-stop) or positive-strand HIV-1 cDNAs per ng of schistosome gDNA.

### Quantitative retrotransposon-anchored PCR (qRAP) to detect integrated provirus

Total genomic DNA from schistosomula exposed to active or heat-inactivated virions and harvested 24 or 48 hours after transduction was isolated as above. Based on the *Alu*-PCR approach to quantify the copy number of integrated HIV-1 provirus in human cells [22], and on an endogenous retrotransposon-anchored PCR technique (RAP) we have previously employed to identify transposons and proviral transgenes integrated in the genome of *S*. *mansoni* [15, 76], we developed a quantitative anchored PCR-based approach (qRAP), to identify and quantify retrovirus integrations into the schistosome genome [23]. In brief, qRAP includes two consecutive PCRs (Fig 2C); the first, retrotransposon anchored PCR (RAP), is a multiplex PCR using a specific primer for the *gag* gene of HIV-1 in tandem with primers specific for endogenous retrotransposons present at high copy number and apparently interspersed throughout the genome of natural populations of *S*. *mansoni* [6, 77]. Second, RAP products are used as template for quantitative PCRs, targeting the LTR sequence of HIV-1. The RAP was performed using 100 ng template gDNA from populations of active-, heat-inactivated- lentivirus-transduced schistosomes or control untreated parasites, Platinum *Taq* DNA Polymerase High Fidelity (Invitrogen) and primers specific for the retrotransposons *SR1*, *SR2*, *fugitive*, *Boudicca* and *SMα* in combination with the *gag*-specific primer in a 50 μl reaction. Two primer mixes were used: mix 1: *SR1*F (200 nM), *SR1*R (200 nM) *SR2*F (200 nM), *SR2*R (200 nM) and *gag* (1.2 μM); mix 2: *fugitive* F (200 nM), *fugitive* R (200 nM) *Boudicca* F (200 nM), *SMα* (200 nM) and *gag* (1.2 μM) (S3 Table). RAP cycling conditions were 94°C for 2 min followed by 20 cycles of 94°C for 30 s, 55°C for 30 s and 68°C for 10 min, with a final extension at 68°C for 10 min. RAP products were employed as template in a quantitative PCR targeting *gag* performed as described [23]. S3 Table provides the sequences of the primers and Taqman probe employed in the RAP and qPCR. Quantitative PCRs were performed in triplicate, using 96-well plates (Bio-Rad), with a denaturation step at 95°C of 3 min followed by 40 cycles of 30 sec at 95°C and 30 sec at 55°C, using a real-time thermal cycler (iCycler, Bio-Rad) fitted with the Bio-Rad iQ5 detector. Reactions were carried out in 20 μl volumes with *gag* primer-probe sets, Perfecta qPCR FastMix, UNG (Quanta Bioscience, Gaithersburg, MD), and as template, 5 μl of the RAP amplicons (diluted 1 in 10) or matched dilutions of non-preamplified samples, i.e., dilutions of gDNA that were not amplified by RAP. Quantification was undertaken using copy number standards, as above. LTR copy number was estimated by interpolation of the PCR signals from a standard curve [78]. LTR copy numbers from schistosomes exposed to virions are presented as fold-increase of RAP-preamplified copy number compared to the non-preamplified copy number or relative copy number of provirus, i.e. copy number of RAP-preamplified qPCR products divided by copy number of non-preamplified qPCR products [23]. The PCR efficiency for the primers/probe set was estimated to be 100 ± 5% by titration analysis [78]

### Inhibitors of reverse transcriptase and integrase

Two reverse transcriptase inhibitors, the nucleoside analogue azidothymidine (AZT) and the non-nucleoside analogue nevirapine (NVP), and the integrase inhibitor 118-D-24, C_11_H_9_N_3_O_4_ [79] were employed to pre-treated schistosomula 24 hours before exposed to pseudotyped HIV-1 virions. Schistosomula were exposed to 10 μM AZT, 10–50 μM NVP, 100 μM of 118-D-24 or corresponding vehicle controls, spinoculated in the presence of VSV-G pseudotyped-HIV-1 virions (above), for 24 and/or 48 hours after which genomic DNA was isolated from the drug-exposed worms. Reduction in the integrated-provirus copy number was not detected using qRAP following exposure to inhibitors of reverse transcriptase or integrase compared to controls (S3 Fig). These findings suggested that schistosomes, unlike mammalian cells, may not activate the drugs to toxic forms, and/or that schistosome cells pump out the drugs quickly [58] or indeed that the drugs did not enter the schistosomes, so that the HIV-1 enzymes were not inhibited. In overview, however, given both the similarities and dissimilarities between human cells and schistosomes in the ability to support reverse transcription and integration and the dissimilarities in the effects of these three inhibitors, there likely exist differences in the physiology of the schistosome versus human cells in regard to the HIV-1 developmental cycle.

### Orthologues of cellular co-factors of reverse transcription and pre-integration complexes

The amino acid sequences of human host cellular factors associated with HIV-1 reverse transcription and pre-integration complexes during the upstream events of the retrovirus life cycle were employed as queries in Blastp searches of the public databases http://blast.ncbi.nlm.nih.gov/Blast.cgi including the draft genome version 5.0 of *S*. *mansoni*, aiming to identify schistosome orthologues/ homologues that might be capable of interact with HIV-1 during the infection, reverse-transcription and provirus integration. Several tentative candidates, including cyclophilin A and importin-α3,4 involved in the HIV capsid binding and nuclear translocation of reverse transcription complexes, respectively, were identified with identity percentages ranging from 23% to 64% (S5 Table). The presence of predicted schistosome homologues of human factors associated with HIV-1 reverse transcription and pre-integration complexes may explain why VSV-G-HIV-1 virions infect schistosome cells and complete the reverse-transcription and integration of the provirus in the genome of the transduced parasite. However, further studies, including the identification, cloning and functional characterization of these schistosome factors, are needed.

### Whole genome shotgun libraries

Total genomic DNA (1,700 ng) isolated from lentivirus-transfected schistosomula as described above, was used directly for preparation of amplification-free 200–400 bp paired end Illumina libraries using a protocol based on a previously described method [80] but using Agencourt AMPure XP beads for sample clean up and size selection. DNA was precipitated onto beads after each enzymatic stage with an equal volume of 20% polyethylene glycol 6000 and 2.5 M NaCl. Beads were not separated from the sample throughout the process until after the adapter ligation stage, after which new beads were used for size selection. This library was sequenced directly as a Whole Genome Sequencing Library (WGS) (S1 Table) without being subjected to TraDIS, as described below (Illumina sequencing).

### Modified Transposon Directed Insertion-site Sequencing (TraDIS) libraries

Two hundred ng of genomic DNA prepared from schistosomula exposed to pseudotyped HIV-1-virions was used to prepare an Illumina library, as described above, but using double stranded Splinkerette V1.2 adapters formed by annealing the oligonucleotide ‘Splinkerette V1.2 top’ G*TTCCCATGGTACTACTCATATAATACGACTCACTATAGGTGACAGCGAGCGC*T (the asterisk indicates phosphorothioate; phosphorothioate linkages resist nuclease degradation [81]) and the oligonucleotide ‘Splinkerette V1.2 bottom’, G*CGCTCGCTGTCACCTATAGTGAGTCGTATTATAATTTTTTTTTCAAAAAA*A. Adapter-ligated fragments, 423 ng, were employed for the amplification of the 3’- or 5’-termini of the sites of integration of HIV-1 into the schistosome genome. Nested oligos, detailed in S2 Table, were used to amplify the 3’- and 5’-ends of integrated HIV-1 proviruses using the Kapa Hifi Hotstart Ready mix. The thermal cycles of the first PCR, ‘PCR1’, comprised denaturation at 95°C, followed by 18 cycles of 98°C, 20 sec, 58°C, 20 sec and 72°C, sec, and concluded with 72°C for 5 minutes. The second PCR, ‘PCR2’, commenced using 24 μl of ‘PCR1’ (after thermocycling), followed by the conditions as for ‘PCR1’ with 12 (rather than 18) cycles of 98°C, 20 sec. S7 Fig presents a schematic of the construction plan for the Transposon Directed Insertion-site Sequencing (TraDIS) libraries from schistosomula exposed to pseudotyped HIV-1 virions.

### Illumina sequencing

Libraries were denatured using 100 mM NaOH and diluted to 6 pM in a hybridization buffer to allow the template strands to hybridize to adapters immobilized on the surface of the flow cell. Cluster amplification was performed in an Illumina cBOT using the V3 cluster generation kit. Thereafter a SYBRGreen QC was performed to measure cluster density and determine whether to pass or fail the flow cell for sequencing, followed by linearization, blocking and hybridization of the R1 sequencing primer. The hybridized flow cells were loaded onto a HiSeq 2000 for 100 cycles of sequencing-by-synthesis using the V3 SBS sequencing kit. Subsequently, the linearization, blocking and hybridization step was repeated in situ to regenerate clusters, release the second strand for sequencing and to hybridize the R2 sequencing primer followed by another 100 cycles of sequencing to produce paired end reads. These steps were performed using proprietary reagents according to the manufacturer's recommendations, https://icom.illumina.com/. The RTA1.8 analysis pipelines were employed to analyze data obtained from the Illumina HiSeq instrument (S1 Table).

Generating TraDIS libraries followed the above method, modified as follows. First, the reaction mix was spiked with 30% phiX to increase nucleotide diversity (PhiX Control v3, catalogue no. FC-110-3001, Illumina, San Diego, CA). Second, the forward primer was specific to the construct (S2 Table) and the reverse primer was Spl_rev_seq (S2 Table). Third, 150 bp paired end reads were produced on a MiSeq instrument.

### Analysis of data—modified TraDIS and WGS

Illumina reads produced from the whole genome sequences were aligned to the reference genome of *S*. *mansoni* [6] and to plasmid pNL4-3, which includes the entire sequence of HIV-1 (GenBank AF324493.1) in parallel using SMALT, http://www.sanger.ac.uk/resources/software/smalt/. Reads that aligned to both references were checked manually for false positives, and the integration positions investigated (S4 Table). Representative false positive integrations, and events that lacked of strong evidence of integration are indicated in S4 Table, bottom table. For modified TraDIS sequence analysis, as the expected DNA fragment was around ~300 bp, FLASH [82] was first run to locate overlap between read pairs. Read pairs were merged into a single read for subsequent analysis if there was ≥10 bp overlap. After a round of quality control to remove PCR and splinkerette adaptors at sequence ends, four categories of reads remained for closer investigation: 5’-merged, 5’-paired reads, 3’-merged and 3’-paired reads. These reads were aligned to the reference genomes of *S*. *mansoni* and to HIV-1, as above. Integrations were considered to be authentic if: i) the Illumina sequence began with the lentivirus (for 3’-library CTCAGACCCTTTTAGTCAGTGTGGAAAATCTCTAGCA corresponding the 3’- 37 bp of LTR; for 5’-library TTGTCTTTTTTGGGACCAAATTAGCCCTTCCA corresponding the 3’-32 bp of LTR); ii) the start of the sequence was not immediately followed by splinkerette adaptor sequences; iii) the remainder of the sequence uniquely mapped to ≥ 30 bp the *S*. *mansoni* reference; and iv) the mapping quality was Q30, corresponding an alignment error rate of 0.1%. PCR duplicate mappings were deleted. Multiple matches within 250 bp of each other were classified a single, unique match in the genome assembly. We categorized the integration clusters as exon, intron, and intergenic regions (Fig 5A) based on annotation of the genome of *S*. *mansoni* [6] in GeneDB, http://www.genedb.org/Homepage/Smansoni.

The confidence interval for the number of integrations in a cell was calculated for the binomial proportion of successful events p = 60 / 207,576,406. Here, 207,576,406 is the number of properly placed paired reads from the sequencing run, which is equivalent to number of sequenced genomic 100-base segments from which an integration (if present) would be detectable. The frequency was scaled using the number of segments per diploid genome and, using the R library binom and the “exact” method; confidence intervals were calculated for a confidence limit of 0.95.

### Accession numbers

Sequence data generated here are available at the European Nucleotide Archive (ENA) accession number ERP002117, http://www.ebi.ac.uk/ena/data/view/ERP002117.

## Supporting Information

S1 Fig Localization of the vesicular stomatitis virus glycoprotein (VSVG) pseudotyped HIV-1 virions on the surface of the adult stages of *Schistosoma mansoni*. **Panels A, B**. Bright and fluorescent field, respectively, of female adult worm exposed to virions for three hours, fixed and probed with secondary antibody only (control). **Panels C-F**. Representative pictures of female adult worms exposed to virions for one (C, D) or three hours (E, F), fixed and probed sequentially with primary and secondary antibodies. Bright and fluorescence fields shown in panels C, E and D, F, respectively. Micrographs captured with the same exposure and magnification, 10X. Scale bar, 100 μm
(PPTX)

S2 Fig Localization of the vesicular stomatitis virus G protein (VSV-G) on the surface of the schistosomula of *Schistosoma mansoni* exposed to VSV-G-pseudotyped HIV-1. Control, non-virion-exposed schistosomulum, and virion-exposed schistosomula harvested at 30 min, one h, two h and three h after exposure, respectively, as indicated. All images were captured with the same exposure time and same magnification (40x). Scale bar = 100μm. Fluorescence intensity quantified by ImageJ (bottom, right), arbitrary units: ratio between parasite signal intensity and background. One-way ANOVA among groups *P* ≤ 0.01, Tukey test between indicated group and control: *P ≤ 0.05, ** *P* ≤ 0.01. Bar, standard error of the mean, n = 10.
(PPTX)

S3 Fig Inactivity of inhibitors of reverse transcriptase and integrase. **Panel A**. Detection of integrated HIV-1 provirus in schistosomula pre-treated with the reverse transcriptase inhibitor azidothymidine (+AZT) or vehicle control (-AZT) for 24 hours, exposed to VSVG-HIV-1 isolate NL4-3, and harvested 24 and 48 hours later for qRAP analysis. **Panel B**. Real-time RCR quantitation of positive strand HIV-1 cDNA in schistosomules inoculated with VSVG-pseudotyped HIV-1 and treated with 10 μM nevirapine, 24 hours after inoculation (bars: standard deviation (SD) of eight independent measurements). **Panel C.** Detection of integrated HIV-1 provirus in schistosomula pre-treated with the reverse transcriptase inhibitor nevirapine (+NVP) or vehicle control (-NVP) for 24 hours, exposed to VSVG-HIV-1, and harvested 24 hours later for qRAP analysis. **Panel D**. Measurement of HIV-1 capsid p24 protein by ELISA in culture media of human Hep-G2 cells infected with the same VSVG-pseudotyped HIV-1 NL4-3 and treated with indicated concentrations of AZT and NVP, 72 hours after infection (bars: standard deviation (SD) of three independent measurements). **Panel E.** Detection of integrated HIV-1 provirus in schistosomula pre-treated with integrase inhibitor 118D24 (+118-D-24) or vehicle control (-118-D-24) for 24 hours before exposure to VSV-G-HIV-1; worms retrieved 24 hours later for qRAP, using RAP primer sets numbers 1 and 2, specific for endogenous mobile genetic elements *SR1* and *SR2* (set 1), and for *fugitive*, *SMα*, and *Boudicca*, respectively (set 2). Findings displayed in panels A, C and E represent the outcome of duplicated experiments; repeat assays used different batches of virions, and similar outcomes were obtained in each repeat.
(PPTX)

S4 Fig HIV-1 integration junctions recovered by modified Transposon Directed Insertion-site Sequencing (TraDIS). Multiple sequence alignment of HIV integrase-driven integration events identified in the 3’-end LTR library; the red triangle indicates the integration boundary. Asterisk: sequence read shown in Fig 3C.
(PPTX)

S5 Fig HIV-1 integration events detected by whole genome sequencing (WGS) of VSV-G HIV-1-transduced schistosomula. **Panel A**. The presence of HIV-1 integration events into the genome of *S*. *mansoni* was determined by identifying two read mapping scenarios; (1)‘partial’ read pairs, where a single read aligned both to the *S*. *mansoni* reference genome and to the HIV-1 reference; 35 integrations of this type were located; and 2) ‘independent’ pairs, where one of the read pair aligned solely to the *S*. *mansoni* reference and the other solely to the HIV reference; 25 of these were identified. Red and blue arrows indicate reads that aligned to schistosome or HIV-1 genome, respectively. The blue line denotes the sequence segment that aligned to HIV that is adjacent to a schistosome segment (red arrow) in this example of a ‘partial’ scenario. Details of the alignments for the two scenarios are shown in S4 Table. **B**. Representative alignment of a read to genomes of HIV-1 and *S*. *mansoni*, identifying a HIV-1 integration within the ZW sex chromosome.
(PPTX)

S6 Fig ‘Spinoculation’ enhances transduction of schistosomula by VSV-G-pseudotyped HIV-1 virions, leading to increased numbers of integrations of proviral HIV-1 into chromosomes. **Panel A.** Quantitation of the HIV-1 cDNA in DNA of schistosomula at 24 h and 48 h after spinoculation or regular transfection with active lentivirus virions. **B.** Detection by qRAP of HIV-1 provirus in the schistosome genomic DNA using the primer set #1 containing specific primers for *SR1* and *SR2* retrotransposons. **C.** Detection by qRAP of HIV-1 provirus in the schistosome genomic DNA using the primer set #2 containing primers specific for the *fugitive*, *SM alpha*, and *Boudicca* transposable elements. Statistical analysis: Student’s *t*-test; *, **—*P* ≤ 0.05, *P* ≤ 0.01 (active vs. heat-inactivated virions). The experiments were triplicated.
(PPTX)

S7 Fig Construction of Transposon Directed Insertion-site Sequencing (TraDIS) libraries from HIV virion transduced schistosomes. Schematic representation of a representative HIV-1 provirus integrated into the gDNA isolated from HIV-transduced parasites. The HIV provirus genome is flanked by the 634 bp long terminal repeats (LTRs) at the 5’-end (5’LTR) and 3’-termini (3’LTR). Mechanical fragmentation of the genomic DNA was followed by repair of the fragment ends, adenylation, ligation of the Illumina adapters, and two rounds of semi-nested PCR; colored primers represent the primer used for the second PCR and also for sequencing–the 3’end of the 5’LTR sequencing primer in blue and the 3’end of the 3’LTR sequencing primer in red annealed 32 bp and 37 bp away from the end of the 5’LTR and 3’LTR, respectively. The 32 bp and 37 bp sequences at the end of the 5’LTR and 3’LTR, respectively, are shown in S2 Fig) A size selection and bead purification of the 5’LTR-end and 3’LTR-end libraries was performed. The fragment selected from 200 bp to 400 bp was employed to construct the libraries. The purified libraries were quantified by qPCR and loaded into Illumina flow cells. Map not to scale.
(PPTX)

S1 Table Summary of Illumina sequencing libraries for 1) modified Transposon Directed Insertion-site Sequencing (TraDIS), the 3’- and the 5’-LTR libraries, and 2) Whole Genome Sequencing (WGS) approaches. (XLSX)

S2 Table Sequences of oligonucleotides for modified TraDIS libraries (3’- and 5’-LTR libraries). (XLSX)

S3 Table Primer sequences for quantitative Retrotransposon Anchored PCR (qRAP)–RAP primers for the end-point PCR, and primers and Taqman probe for the qPCR. (XLSX)

S4 Table WGS data with first and second Illumina reads that mapped to HIV-1 and *Schistosoma mansoni* genomes. (XLSX)

S5 Table Orthologues/ homologues in the genome of *Schistosoma mansoni* of cellular components that associate in human cells with HIV-1 reverse transcription and pre-integration complexes. (XLSX)
