# Supplementary figures and images for: HIV-1 Integrates Widely throughout the Genome of the Human Blood Fluke Schistosoma mansoni
Source: PLoS Pathog. 2016 Oct 20;12(10):e1005931. doi: 10.1371/journal.ppat.1005931 (PMC5072744; doi:10.1371/journal.ppat.1005931)

## Slide 1
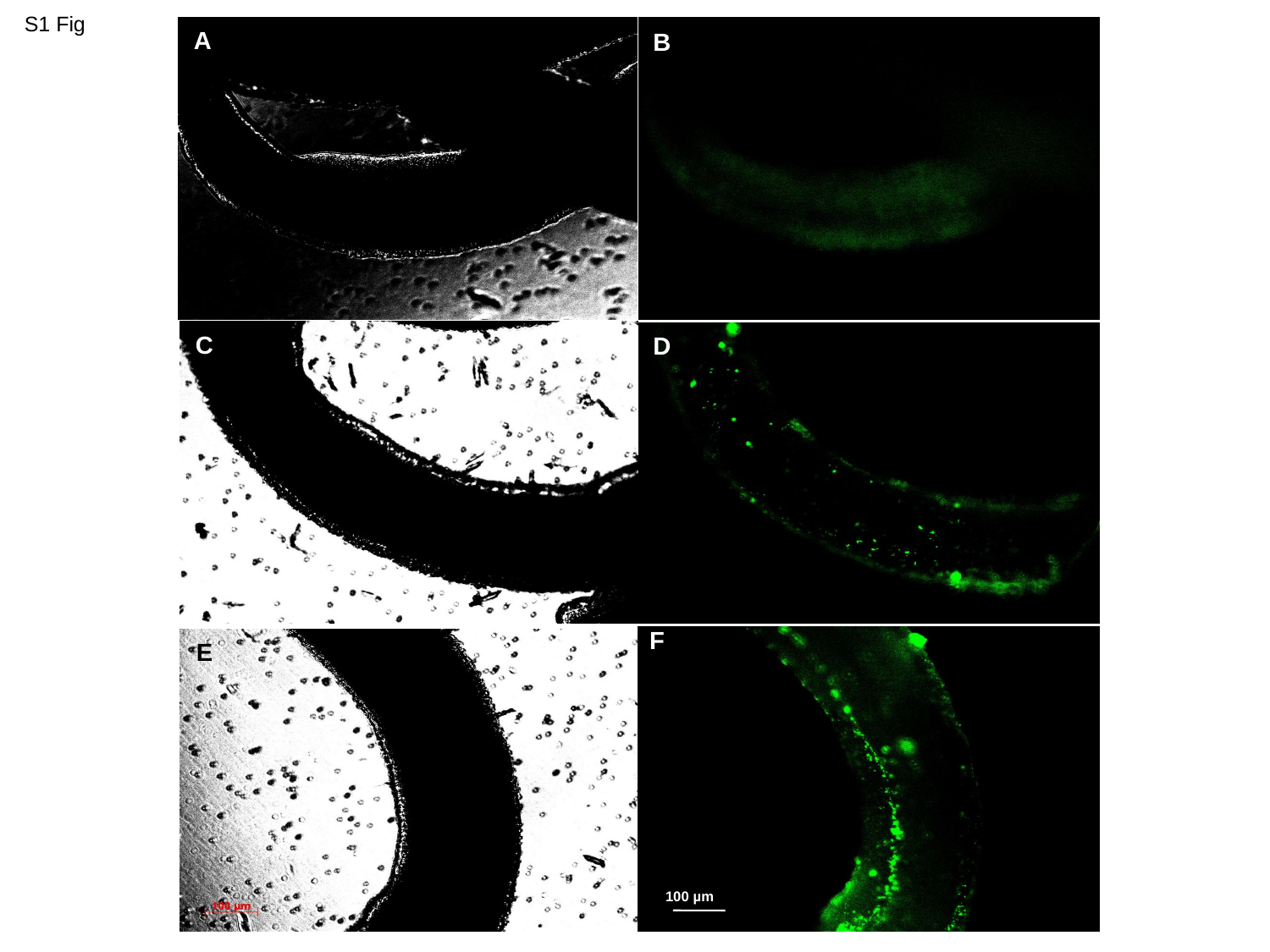

S1 Fig
D
B
A
B
F
C
D
G
F
F
E
100 µm

Supplement: S1 Fig — Panels A, B. Bright and fluorescent field, respectively, of female adult worm exposed to virions for three hours, fixed and probed with secondary antibody only (control). Panels C-F. Representative pictures of female adult worms exposed to virions for one (C, D) or three hours (E, F), fixed and probed sequentially with primary and secondary antibodies. Bright and fluorescence fields shown in panels C, E and D, F, respectively. Micrographs captured with the same exposure and magnification, 10X. Scale bar, 100 μm (PPTX) [file ppat.1005931.s001.pptx]

## Slide 1
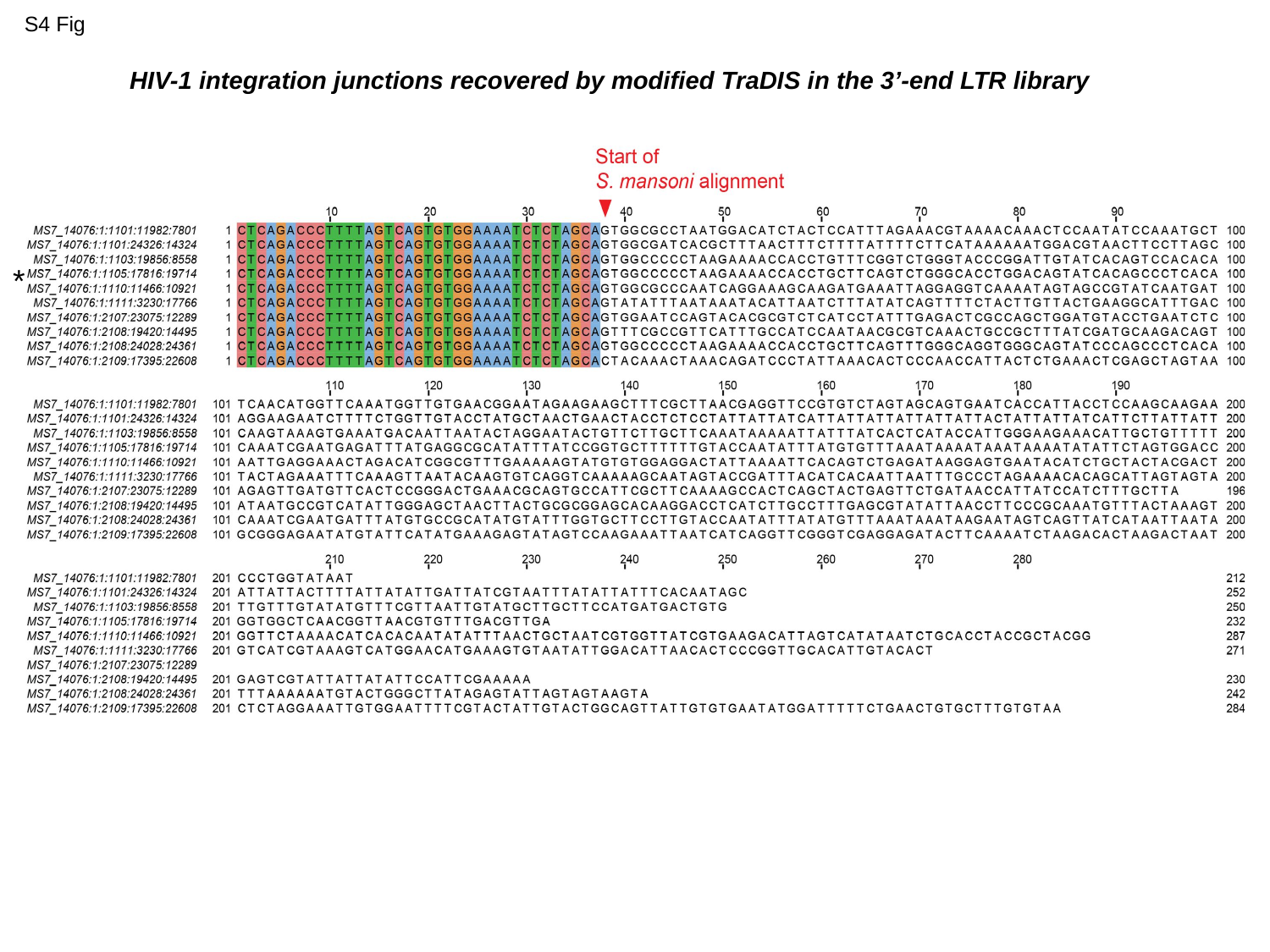

S4 Fig
HIV-1 integration junctions recovered by modified TraDIS in the 3’-end LTR library
*

Supplement: S4 Fig — Multiple sequence alignment of HIV integrase-driven integration events identified in the 3’-end LTR library; the red triangle indicates the integration boundary. Asterisk: sequence read shown in Fig 3C. (PPTX) [file ppat.1005931.s004.pptx]
