# Supplementary material for: HIV-1 Integrates Widely throughout the Genome of the Human Blood Fluke Schistosoma mansoni
Source: PLoS Pathog. 2016 Oct 20;12(10):e1005931. doi: 10.1371/journal.ppat.1005931 (PMC5072744; doi:10.1371/journal.ppat.1005931)
Supplement: S2 Fig — Control, non-virion-exposed schistosomulum, and virion-exposed schistosomula harvested at 30 min, one h, two h and three h after exposure, respectively, as indicated. All images were captured with the same exposure time and same magnification (40x). Scale bar = 100μm. Fluorescence intensity quantified by ImageJ (bottom, right), arbitrary units: ratio between parasite signal intensity and background. One-way ANOVA among groups P ≤ 0.01, Tukey test between indicated group and control: *P ≤ 0.05, ** P ≤ 0.01. Bar, standard error of the mean, n = 10. (PPTX) [file ppat.1005931.s002.pptx]

## Slide 1
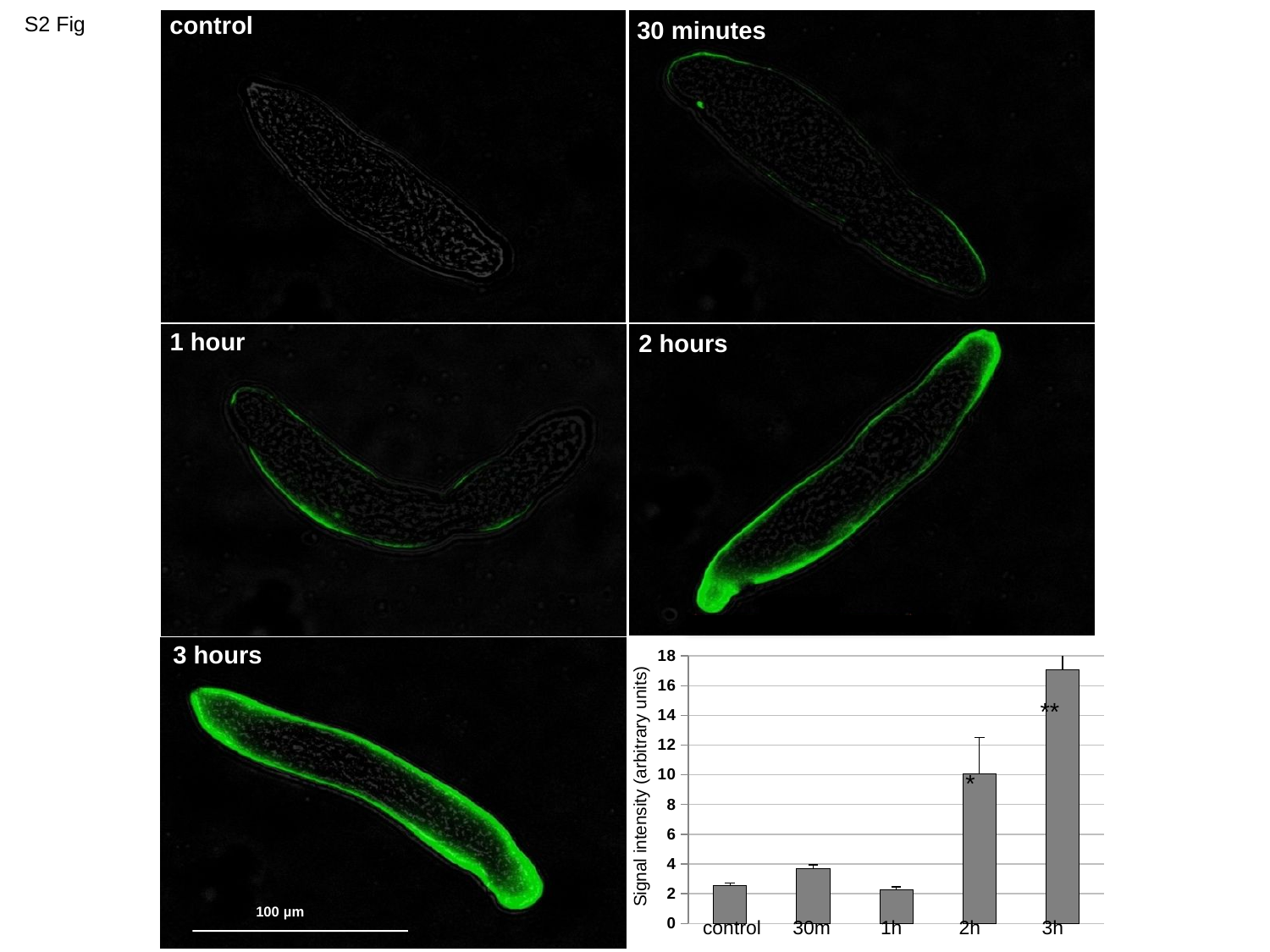

control
30 minutes
D
1 hour
2 hours
F
3 hours
100 µm
### Chart
| Category | |
|---|---|
| A | 2.542525131880163 |
| C | 3.706116831965074 |
| D | 2.277098678883864 |
| E | 10.05544729027468 |
| F | 17.09175110019445 |control
30m
1h
2h
3h
**
*
Signal intensity (arbitrary units)
S2 Fig
