# Supplementary material for: HIV-1 Integrates Widely throughout the Genome of the Human Blood Fluke Schistosoma mansoni
Source: PLoS Pathog. 2016 Oct 20;12(10):e1005931. doi: 10.1371/journal.ppat.1005931 (PMC5072744; doi:10.1371/journal.ppat.1005931)
Supplement: S3 Fig — Panel A. Detection of integrated HIV-1 provirus in schistosomula pre-treated with the reverse transcriptase inhibitor azidothymidine (+AZT) or vehicle control (-AZT) for 24 hours, exposed to VSVG-HIV-1 isolate NL4-3, and harvested 24 and 48 hours later for qRAP analysis. Panel B. Real-time RCR quantitation of positive strand HIV-1 cDNA in schistosomules inoculated with VSVG-pseudotyped HIV-1 and treated with 10 μM nevirapine, 24 hours after inoculation (bars: standard deviation (SD) of eight independent measurements). Panel C. Detection of integrated HIV-1 provirus in schistosomula pre-treated with the reverse transcriptase inhibitor nevirapine (+NVP) or vehicle control (-NVP) for 24 hours, exposed to VSVG-HIV-1, and harvested 24 hours later for qRAP analysis. Panel D. Measurement of HIV-1 capsid p24 protein by ELISA in culture media of human Hep-G2 cells infected with the same VSVG-pseudotyped HIV-1 NL4-3 and treated with indicated concentrations of AZT and NVP, 72 hours after infection (bars: standard deviation (SD) of three independent measurements). Panel E. Detection of integrated HIV-1 provirus in schistosomula pre-treated with integrase inhibitor 118D24 (+118-D-24) or vehicle control (-118-D-24) for 24 hours before exposure to VSV-G-HIV-1; worms retrieved 24 hours later for qRAP, using RAP primer sets numbers 1 and 2, specific for endogenous mobile genetic elements SR1 and SR2 (set 1), and for fugitive, SMα, and Boudicca, respectively (set 2). Findings displayed in panels A, C and E represent the outcome of duplicated experiments; repeat assays used different batches of virions, and similar outcomes were obtained in each repeat. (PPTX) [file ppat.1005931.s003.pptx]

## Slide 1
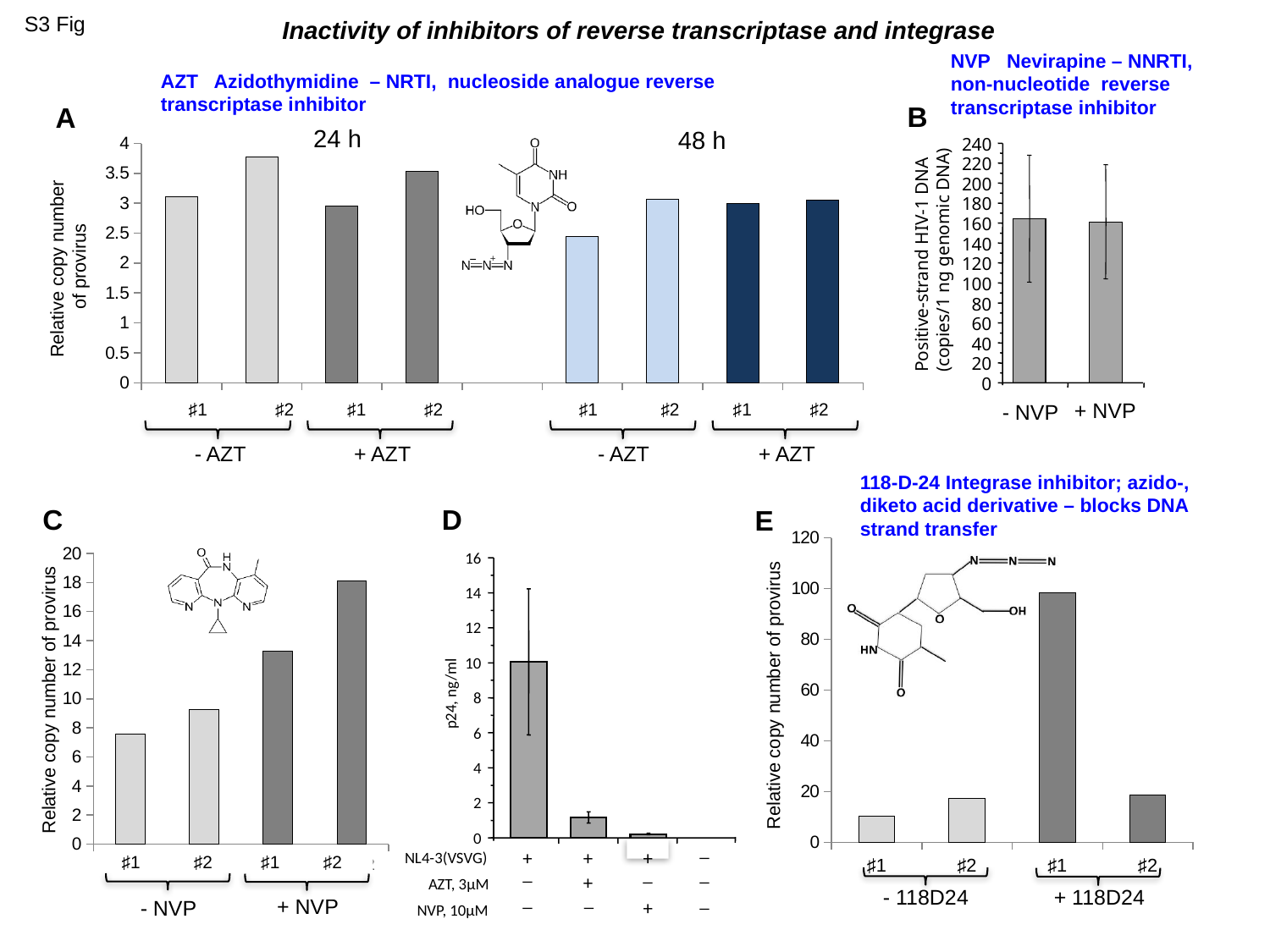

S3 Fig
Inactivity of inhibitors of reverse transcriptase and integrase
NVP Nevirapine – NNRTI, non-nucleotide reverse transcriptase inhibitor
AZT Azidothymidine – NRTI, nucleoside analogue reverse transcriptase inhibitor
B
A
24 h
48 h
### Chart
| Category | |
|---|---|
240
220
200
180
160
140
Positive-strand HIV-1 DNA
(copies/1 ng genomic DNA)
Relative copy number
of provirus
120
100
80
60
40
20
0
+ NVP
 ♯1 ♯2 ♯1 ♯2 ♯1 ♯2 ♯1 ♯2
- NVP
- AZT
+ AZT
- AZT
+ AZT
118-D-24 Integrase inhibitor; azido-, diketo acid derivative – blocks DNA strand transfer
C
D
### Chart
| Category | |
|---|---|
| ♯1 | 10.45000000000001 |
| ♯2 | 17.36 |
| ♯1 | 98.19 |
| ♯2 | 18.67000000000001 |Relative copy number of provirus
- 118D24
+ 118D24
E
### Chart
| Category | |
|---|---|
| mix ♯1 | 7.55 |
| mix ♯2 | 9.280000000000001 |
| mix ♯1 | 13.28 |
| mix ♯2 | 18.12 | ♯1 ♯2 ♯1 ♯2
16
14
12
10
p24, ng/ml
8
6
4
2
0
_
+
+
+
NL4-3(VSVG)
_
_
_
+
AZT, 3µM
_
_
_
+
NVP, 10µM
Relative copy number of provirus
+ NVP
- NVP
