# Supplementary material for: HIV-1 Integrates Widely throughout the Genome of the Human Blood Fluke Schistosoma mansoni
Source: PLoS Pathog. 2016 Oct 20;12(10):e1005931. doi: 10.1371/journal.ppat.1005931 (PMC5072744; doi:10.1371/journal.ppat.1005931)
Supplement: S5 Fig — Panel A. The presence of HIV-1 integration events into the genome of S. mansoni was determined by identifying two read mapping scenarios; (1)‘partial’ read pairs, where a single read aligned both to the S. mansoni reference genome and to the HIV-1 reference; 35 integrations of this type were located; and 2) ‘independent’ pairs, where one of the read pair aligned solely to the S. mansoni reference and the other solely to the HIV reference; 25 of these were identified. Red and blue arrows indicate reads that aligned to schistosome or HIV-1 genome, respectively. The blue line denotes the sequence segment that aligned to HIV that is adjacent to a schistosome segment (red arrow) in this example of a ‘partial’ scenario. Details of the alignments for the two scenarios are shown in S4 Table. B. Representative alignment of a read to genomes of HIV-1 and S. mansoni, identifying a HIV-1 integration within the ZW sex chromosome. (PPTX) [file ppat.1005931.s005.pptx]

## Slide 1
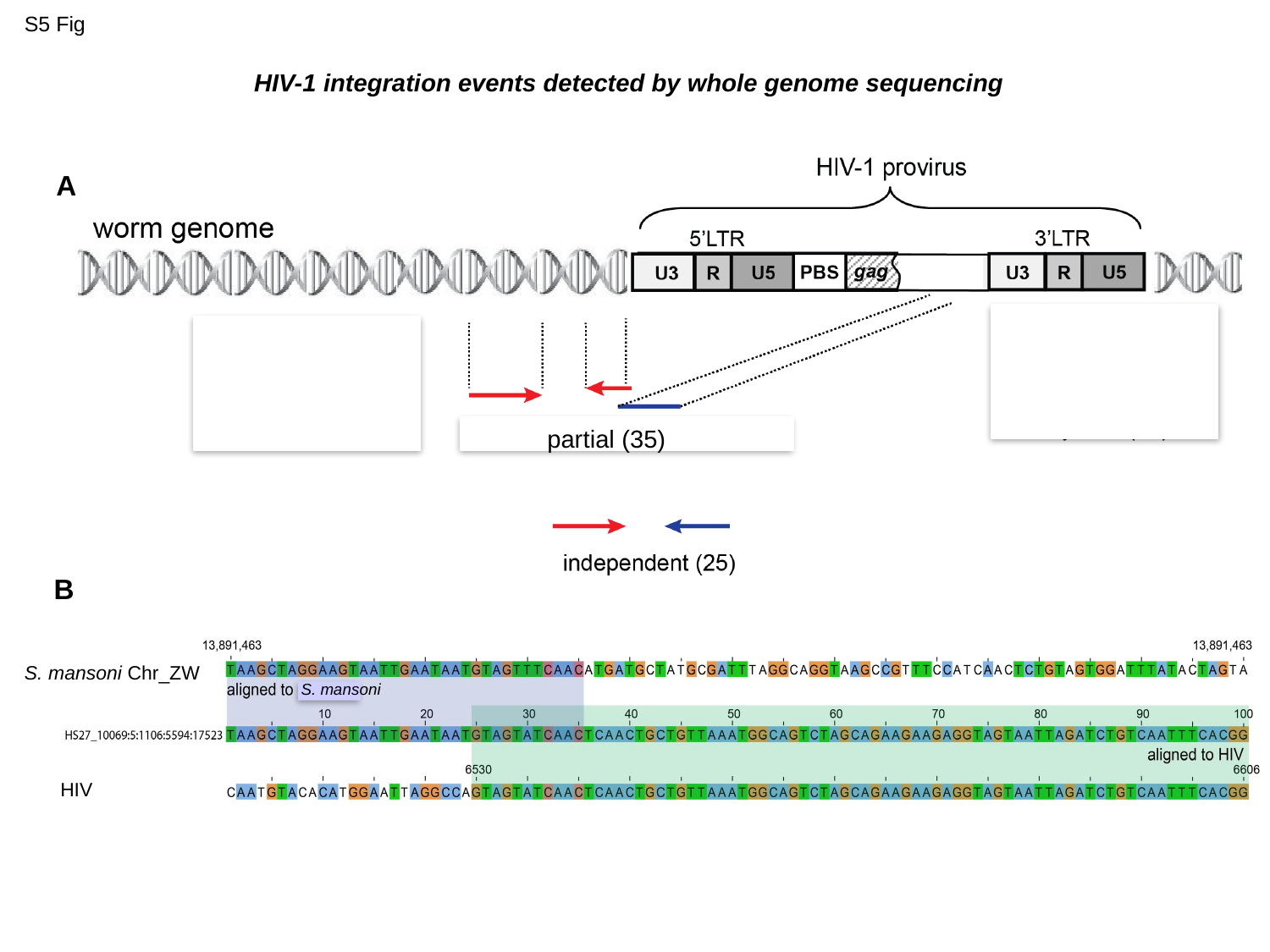

S5 Fig
HIV-1 integration events detected by whole genome sequencing
A
B
S. mansoni Chr_ZW
S. mansoni
HIV
partial (35)
