# Supplementary material for: HIV-1 Integrates Widely throughout the Genome of the Human Blood Fluke Schistosoma mansoni
Source: PLoS Pathog. 2016 Oct 20;12(10):e1005931. doi: 10.1371/journal.ppat.1005931 (PMC5072744; doi:10.1371/journal.ppat.1005931)
Supplement: S6 Fig — Panel A. Quantitation of the HIV-1 cDNA in DNA of schistosomula at 24 h and 48 h after spinoculation or regular transfection with active lentivirus virions. B. Detection by qRAP of HIV-1 provirus in the schistosome genomic DNA using the primer set #1 containing specific primers for SR1 and SR2 retrotransposons. C. Detection by qRAP of HIV-1 provirus in the schistosome genomic DNA using the primer set #2 containing primers specific for the fugitive, SM alpha, and Boudicca transposable elements. Statistical analysis: Student’s t-test; *, **—P ≤ 0.05, P ≤ 0.01 (active vs. heat-inactivated virions). The experiments were triplicated. (PPTX) [file ppat.1005931.s006.pptx]

## Slide 1
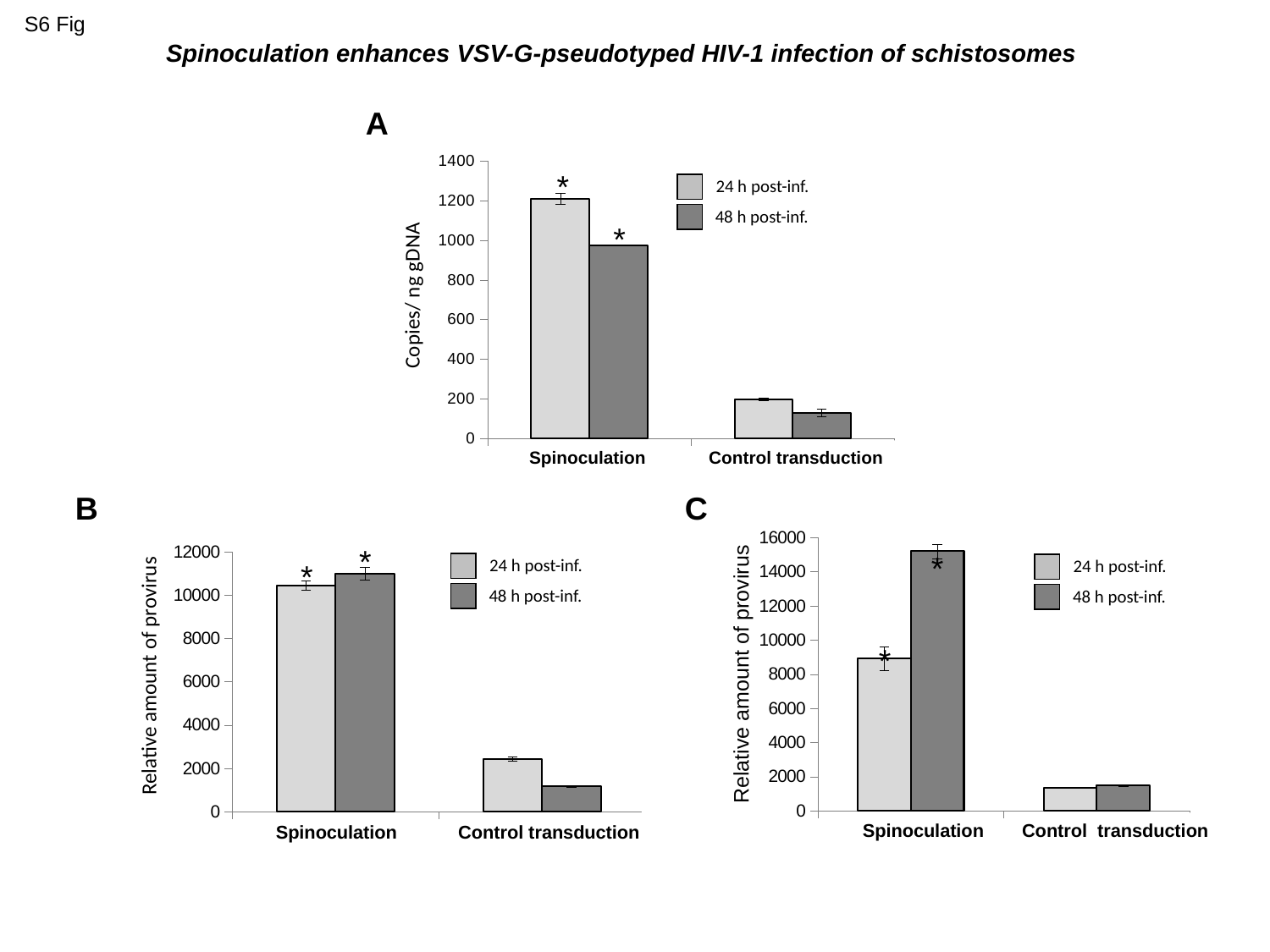

S6 Fig
Spinoculation enhances VSV-G-pseudotyped HIV-1 infection of schistosomes
A
### Chart
| Category | | |
|---|---|---|*
24 h post-inf.
48 h post-inf.
*
Copies/ ng gDNA
Spinoculation
Control transduction
B
### Chart
| Category | | |
|---|---|---|*
*
Relative amount of provirus
Spinoculation
Control transduction
C
### Chart
| Category | | |
|---|---|---|*
*
Relative amount of provirus
Spinoculation
Control transduction
24 h post-inf.
48 h post-inf.
24 h post-inf.
48 h post-inf.
