# Supplementary material for: HIV-1 Integrates Widely throughout the Genome of the Human Blood Fluke Schistosoma mansoni
Source: PLoS Pathog. 2016 Oct 20;12(10):e1005931. doi: 10.1371/journal.ppat.1005931 (PMC5072744; doi:10.1371/journal.ppat.1005931)
Supplement: S7 Fig — Schematic representation of a representative HIV-1 provirus integrated into the gDNA isolated from HIV-transduced parasites. The HIV provirus genome is flanked by the 634 bp long terminal repeats (LTRs) at the 5’-end (5’LTR) and 3’-termini (3’LTR). Mechanical fragmentation of the genomic DNA was followed by repair of the fragment ends, adenylation, ligation of the Illumina adapters, and two rounds of semi-nested PCR; colored primers represent the primer used for the second PCR and also for sequencing–the 3’end of the 5’LTR sequencing primer in blue and the 3’end of the 3’LTR sequencing primer in red annealed 32 bp and 37 bp away from the end of the 5’LTR and 3’LTR, respectively. The 32 bp and 37 bp sequences at the end of the 5’LTR and 3’LTR, respectively, are shown in S2 Fig) A size selection and bead purification of the 5’LTR-end and 3’LTR-end libraries was performed. The fragment selected from 200 bp to 400 bp was employed to construct the libraries. The purified libraries were quantified by qPCR and loaded into Illumina flow cells. Map not to scale. (PPTX) [file ppat.1005931.s007.pptx]

## Slide 1
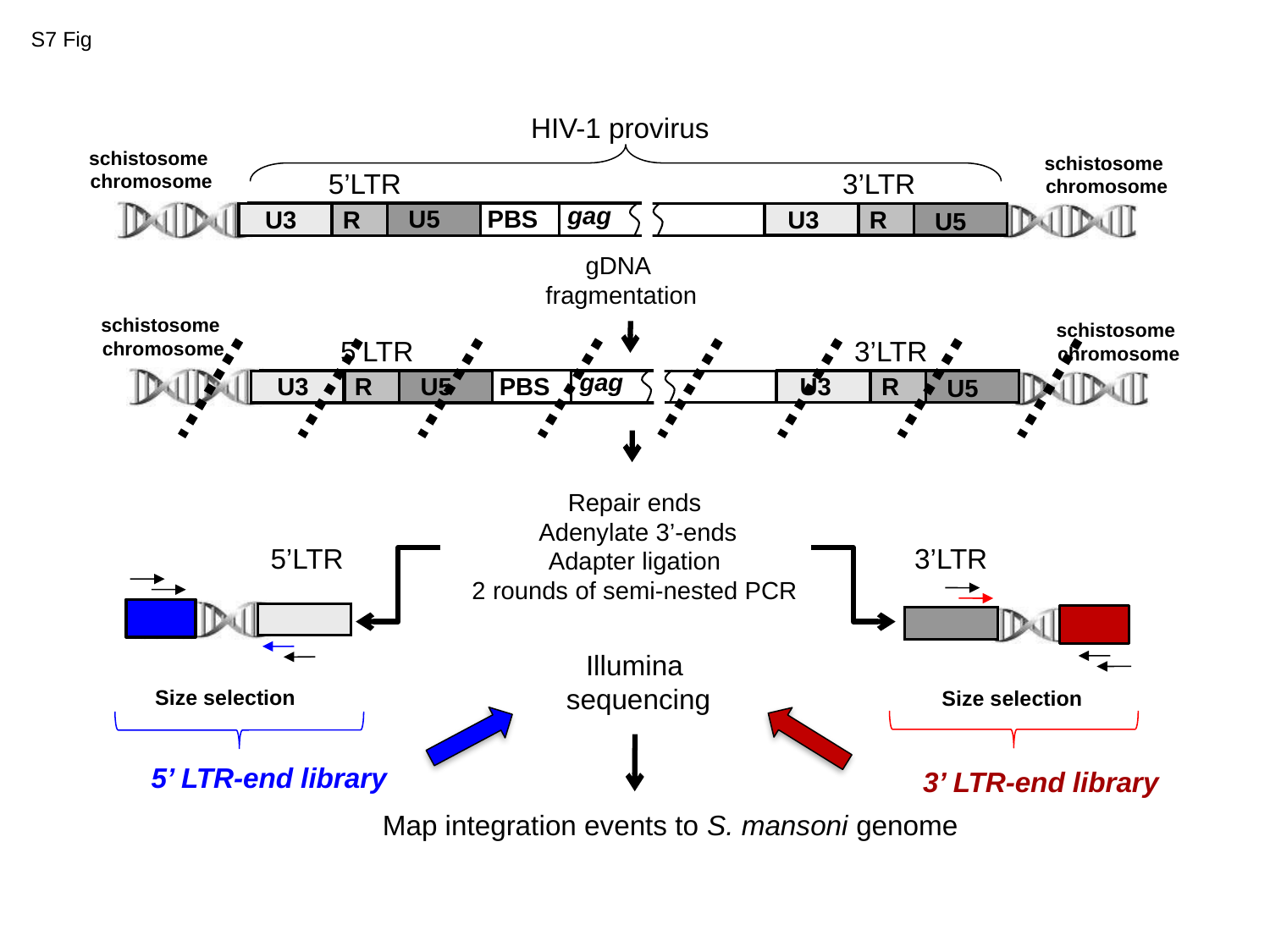

Fig. S7
S7 Fig
HIV-1 provirus
5’LTR
3’LTR
gag
PBS
U5
U3
R
U3
R
U5
schistosome
chromosome
schistosome
chromosome
gDNA
fragmentation
schistosome
chromosome
schistosome
chromosome
5’LTR
3’LTR
gag
PBS
U5
U3
R
U3
R
U5
Repair ends
 Adenylate 3’-ends
Adapter ligation
2 rounds of semi-nested PCR
Illumina
sequencing
Size selection
5’ LTR-end library
Size selection
3’ LTR-end library
Map integration events to S. mansoni genome
5’LTR
3’LTR
